# A Systematic Review of Major Advances in Breast Cancer Therapeutics in 2025: Synthesis of Conference and Published Evidence

**DOI:** 10.3390/ijms27041971

**Published:** 2026-02-19

**Authors:** Nabil Ismaili

**Affiliations:** 1 Department of Medical Oncology, Mohammed VI Faculty of Medicine, Mohammed VI University of Sciences and Health (UM6SS), Casablanca 82403, Morocco; ismailinabil@yahoo.fr or nismaili@um6ss.ma; 2 Mohammed VI Foundation of Sciences and Health (FM6SS), Casablanca 82403, Morocco; 3 Oncopathology, Biology and Environment of Cancer Laboratory, Mohammed VI Center for Research and Innovation (CM6RI), Rabat 11103, Morocco

**Keywords:** breast cancer, systematic review, antibody-drug conjugate, CDK4/6 inhibitor, precision medicine, biomarkers, ASCO 2025, ESMO 2025, SABCS 2025

## Abstract

The year 2025 has been transformative in breast oncology, marked by the maturation of pivotal adjuvant trials, the introduction of novel ADCs, and the validation of proactive biomarker-driven strategies across all molecular subtypes. ASCO, ESMO, and SABCS contributed pivotal updates that further refined treatment paradigms. This systematic review synthesizes and critically evaluates pivotal Phase II/III clinical trials presented at major oncology conferences (ASCO 2025, ESMO 2025, SABCS 2025) and published in high-impact journals during 2025. A curated selection of pivotal Phase II/III trials, and major prospective trials published or presented in 2025 was performed. Data extraction focused on trial design, population, interventions, efficacy endpoints, and safety outcomes. Narrative synthesis was organized by disease stage and molecular subtype. Key 2025 findings (50 clinical trials) include: (1) confirmation of overall survival benefit with adjuvant CDK4/6 inhibitors in HR+/HER2− early breast cancer (monarchE: HR = 0.842, *p* = 0.0273); (2) establishment of trastuzumab deruxtecan (T-DXd) as a new standard in high-risk HER2+ early disease (DESTINY-Breast05: IDFS HR = 0.47) and first-line metastatic settings (DESTINY-Breast09: PFS HR = 0.58); (3) validation of TROP2-directed ADCs as first-line therapy for metastatic triple-negative breast cancer (ASCENT-03: PFS HR = 0.62; BEGONIA: ORR 79%); (4) paradigm shift to proactive, liquid biopsy-guided therapy switching (SERENA-6: PFS HR = 0.44); (5) updated efficacy and safety of the oral SERD imlunestrant from the EMBER-3 trial, supporting its role in ESR1-mutated advanced breast cancer and in combination with abemaciclib; (6) confirmation of long-term survival benefit for neoadjuvant carboplatin in early TNBC and new positive adjuvant data; (7) pivotal advances in HER2+ metastatic disease sequencing with tucatinib and T-DXd; (8) evidence supporting optimized adjuvant endocrine therapy in HER2+/HR+ early disease; and (9) emergence of novel agents with improved therapeutic indices, including PROTAC degraders, oral SERDs, and mutant-selective PI3K inhibitors. The 2025 evidence base has fundamentally reshaped breast cancer management, establishing new standards of care across all subtypes. Unifying themes include biomarker-driven personalization, strategic treatment sequencing, management of unique toxicities, and emphasis on patient-reported outcomes. Future challenges include optimizing treatment integration, managing financial toxicity, and ensuring equitable global access.

## 1. Introduction

### 1.1. The Global Burden and Evolving Landscape of Breast Cancer

Breast cancer maintains its position as the most frequently diagnosed malignancy among women worldwide, with epidemiological projections for 2025 estimating approximately 2.5 million new cases and 700,000 deaths annually [1]. The disease represents not merely a collection of pathological entities but rather a complex spectrum of molecularly defined subtypes with distinct biological behaviors, therapeutic vulnerabilities, and clinical trajectories. The historical demarcation between localized and metastatic disease has become increasingly blurred as therapeutic strategies developed in advanced settings rapidly inform and transform curative-intent approaches. The molecular revolution that began with the identification of hormone receptors and human epidermal growth factor receptor 2 (HER2) has evolved into a sophisticated understanding of genomic alterations, immune microenvironment interactions, and dynamic resistance mechanisms that collectively dictate therapeutic response and disease progression.

The therapeutic odyssey of breast cancer management has progressed through several distinct eras: the empirical use of cytotoxic chemotherapy, the targeted revolution initiated by trastuzumab, the endocrine refinement period marked by aromatase inhibitors and selective estrogen receptor (ER) modulators, and currently what might be termed the “conjugate and combine” era characterized by antibody-drug conjugates (ADCs) and rational combination therapies. Each transition has brought not only improved outcomes but also increased complexity in treatment decision-making, necessitating more nuanced patient stratification and personalized therapeutic approaches [2,3].

### 1.2. 2025: A Watershed Year in Breast Oncology

The year 2025 represents a remarkable inflection point in breast cancer therapeutics, distinguished by several converging developments that collectively redefine standards of care across the disease continuum. This pivotal year is characterized by three fundamental transformations:

First, the maturation of long-term data from trials that initially established therapeutic categories now provides definitive evidence regarding overall survival (OS) benefits and durability of response. The monarchE trial demonstrated a statistically significant OS benefit in early stage disease, while the NATALEE trial showed a sustained invasive DFS benefit and a positive, though not yet statistically significant, trend for OS. Similarly, extended follow-up from pivotal immunotherapy trials in triple-negative breast cancer (TNBC) now provides conclusive evidence regarding long-term survival benefits and late-emerging toxicities.

Second, the therapeutic armamentarium has expanded beyond incremental improvements to include entirely novel mechanisms of action. Proteolysis-targeting chimeras (PROTACs) represent a revolutionary approach to protein degradation, while next-generation selective ER degraders (SERDs) offer oral alternatives to established endocrine therapies. Mutant-selective phosphatidylinositol 3-kinase (PI3K) inhibitors address a long-standing challenge in targeting this pathway by minimizing toxicity associated with the inhibition of the wild-type (non-mutated) PI3K protein, which is widely expressed in healthy tissues. These advances collectively represent not merely new drugs but new therapeutic paradigms.

Third, the integration of biomarker-driven strategies has evolved from static assessment to dynamic monitoring and proactive intervention. The validation of circulating tumor DNA (ctDNA) monitoring to guide therapy switching before radiological progression (as demonstrated in SERENA-6) represents a fundamental shift from reactive to pre-emptive precision medicine. Similarly, refined predictive biomarkers beyond programmed death-ligand 1 (PD-L1) expression are under investigation. For example, stromal tumor-infiltrating lymphocytes (sTILs) show promise as a prognostic and potentially predictive factor in TNBC, but are not yet a validated biomarker to guide clinical decision-making for immunotherapy in routine practice.

### 1.3. The Conference Landscape as a Catalyst for Practice Change

The American Society of Clinical Oncology (ASCO) 2025 Annual Meeting (Chicago, 30 May–3 June), the European Society for Medical Oncology (ESMO) 2025 Congress (Berlin, 17–21 October), and the San Antonio Breast Cancer Symposium (SABCS) 2025 (San Antonio, TX, 9–12 December) served as the principal platforms for unveiling these transformative advances. These conferences have evolved from venues for preliminary data presentation to arenas where practice-defining evidence is simultaneously presented, debated, and contextualized. The convergence of multiple pivotal trials across subtypes created a unique opportunity for cross-disciplinary synthesis and comparative effectiveness assessment that transcends traditional disease stage or molecular classification boundaries.

The simultaneous publication of many conference presentations in high-impact journals (New England Journal of Medicine, The Lancet Oncology, JAMA Oncology, Journal of Clinical Oncology) within weeks of presentation further accelerated the translation of evidence to practice. This synchronous dissemination model has compressed the traditional knowledge translation timeline, creating both opportunities for rapid implementation and challenges for guideline development and healthcare system adaptation.

### 1.4. Rationale and Objectives of This Systematic Review

The rapid pace of innovation, with breakthroughs presented across multiple forums and published in various formats, creates significant challenges for clinicians, researchers, and policymakers attempting to integrate this evidence into coherent practice paradigms. Conference presentations often provide earlier but sometimes incomplete data, while subsequent publications offer more comprehensive analysis but with dissemination delays. This systematic review addresses the critical need to synthesize this dispersed evidence into a coherent, clinically actionable narrative that transcends individual trial results to identify overarching themes, persistent challenges, and future directions.

The primary objectives of this review are fourfold:

1. To systematically identify and evaluate pivotal breast cancer trials presented at major oncology conferences and published in high-impact journals during 2025.
2. To synthesize efficacy and safety data across disease subtypes and clinical settings, with particular attention to practice-changing advances.
3. To extract immediate clinical implications for practice while identifying knowledge gaps requiring further investigation.
4. To identify unifying themes, persistent challenges, and future research priorities that will shape the next phase of breast oncology innovation.

This review is structured to provide both comprehensive detail regarding individual trials and synthesized insights regarding their collective implications for the field. The following sections detail the methodological approach, present results organized by disease stage and molecular subtype, discuss integrated clinical implications, and conclude with recommendations for practice and future research directions.

## 2. Methods

### 2.1. Review Protocol and Framework

This systematic review was conducted according to an a priori established protocol developed in accordance with the Preferred Reporting Items for Systematic Reviews and Meta-Analyses (PRISMA) 2020 statement. While not registered in PROSPERO due to the rapid evidence synthesis timeline required for contemporary clinical guidance, the review employed rigorous methodological standards throughout the selection, evaluation, and synthesis processes. The review framework was designed to balance comprehensiveness with clinical relevance, focusing specifically on evidence with immediate potential to influence practice patterns and treatment guidelines.

### 2.2. Eligibility Criteria

#### 2.2.1. Study Designs

The review included Phase II and Phase III trials as the primary source of evidence. Phase I trials were considered only if they presented pivotal efficacy data with clear practice-changing implications, particularly first-in-human studies demonstrating unprecedented activity in biomarker-selected populations. Single-arm studies were excluded unless they represented the only available evidence for a novel therapeutic agent in a specific clinical context. Retrospective analyses, subgroup analyses of previously published trials, non-randomized prospective studies, and preclinical investigations were excluded to maintain focus on prospective evidence with the highest level of validity for clinical decision-making.

#### 2.2.2. Population

Adult patients (≥18 years) with histologically confirmed breast cancer of any stage were included. Studies focusing exclusively on male breast cancer, pregnancy-associated breast cancer, or specific rare histological subtypes (e.g., phyllodes tumors, angiosarcomas) were excluded unless they addressed therapeutic questions with direct relevance to the broader breast cancer population. Trials including mixed solid tumors were included only if breast cancer-specific outcomes were reported separately (Table 1).

#### 2.2.3. Interventions

Systemic therapies including chemotherapy, endocrine therapy, targeted agents, immunotherapy, antibody-drug conjugates, and novel mechanism agents (PROTAC degraders, bispecific antibodies, etc.) were included. Studies evaluating exclusively surgical techniques, radiotherapy approaches, or supportive care interventions without novel systemic therapy components were excluded. Trials focusing on treatment sequencing, duration, or de-escalation strategies were included given their growing importance in personalized treatment planning (Table 1).

#### 2.2.4. Outcomes

Primary endpoints of interest included OS, progression-free survival (PFS), invasive disease-free survival (IDFS), pCR, and objective response rate (ORR). Secondary endpoints included duration of response, patient-reported outcomes (PROs), quality of life measures, and comprehensive safety profiles. Biomarker analyses were extracted when available, with particular attention to predictive biomarkers for patient selection (Table 1).

#### 2.2.5. Timeframe and Sources

The review focused on evidence presented or published during the 2025 calendar year. Primary sources included:

- Presentations at the ASCO 2025 Annual Meeting (Chicago, 30 May–3 June), including plenary sessions, oral abstract presentations, and late-breaking abstracts.
- Presentations at the ESMO 2025 Congress (Berlin, 17–21 October), including Presidential, Proffered Paper, and late-breaking sessions.
- Presentations at the SABCS 2025 (San Antonio, TX, 9–12 December), including general session, spotlight session, and poster presentations.
- Peer-reviewed publications in journals with an impact factor ≥10 (New England Journal of Medicine, The Lancet Oncology, JAMA Oncology, Journal of Clinical Oncology, Annals of Oncology, Clinical Cancer Research) from 1 January to 31 December 2025.

Conference abstracts subsequently published as full manuscripts were tracked to avoid duplication, with the most comprehensive version used for data extraction.

### 2.3. Search Strategy and Study Selection

#### 2.3.1. Conference Proceedings

The official online programs of ASCO 2025, ESMO 2025 and SABCS were systematically searched using built-in search functions and manual review of session listings. All sessions categorized as “Breast Cancer” or containing breast cancer-related content were reviewed. The late-breaking abstract databases for both conferences were comprehensively examined. Press releases from academic societies, cooperative groups, and pharmaceutical sponsors were screened for announcements of practice-changing data.

#### 2.3.2. Electronic Database Search

Complementary searches of PubMed/MEDLINE and Embase were conducted for the period 1 January 2025, to 31 December 2025. The search strategy employed a combination of controlled vocabulary (MeSH terms, Emtree) and free-text terms related to breast cancer, specific drug classes, and therapeutic approaches. The search was limited to human studies and English language publications.

#### 2.3.3. Study Selection Process 

Identified records from all sources were merged using reference management software (EndNote X20), with automated and manual duplicate removal. Title and abstract screening was performed independently by two reviewers against the eligibility criteria. Discrepancies were resolved through discussion, with adjudication by a third reviewer when necessary. Potentially eligible studies underwent full-text review by the same two independent reviewers. Reasons for exclusion at the full-text stage were documented and categorized. The study selection process followed the PRISMA flow diagram structure, though formal diagram generation was foregone in favor of narrative description given the curated selection approach.

### 2.4. Data Extraction and Management

#### 2.4.1. Data Extraction Form

A standardized data extraction form was developed and pilot-tested on five representative studies. The form captured the following domains:

- Study characteristics: Trial name, NCT identifier, phase, design, funding source, publication/presentation details
- Patient population: Sample size, inclusion/exclusion criteria, molecular subtypes, disease stage, prior therapies, biomarker requirements
- Interventions: Experimental and control regimens, dosing, administration schedules, treatment duration
- Outcomes: Primary and secondary endpoints, efficacy measures with hazard ratios and confidence intervals, safety data including adverse events of special interest
- Biomarker analyses: Pre-specified and exploratory biomarker assessments, predictive and prognostic associations
- Quality assessment domains: Elements relevant to risk of bias assessment

#### 2.4.2. Extraction Process

Study selection was performed independently by two reviewers (Prof. Nabil Ismaili and Prof. Sanaa El Majjaoui). Any disagreements were resolved through discussion; if consensus could not be reached, a third senior reviewer with expertise in breast oncology and systematic review methodology was consulted for final adjudication.

#### 2.4.3. Title and Abstract Screening

All records retrieved from the searches were imported into Covidence systematic review software (https://www.covidence.org/, Veritas Health Innovation, Melbourne, Australia), where duplicates were automatically and manually removed. The two reviewers independently screened the titles and abstracts of all unique records against the eligibility criteria. Records that clearly did not meet criteria (e.g., preclinical study, wrong cancer type) were excluded. All potentially relevant records or those where eligibility was uncertain based on the abstract proceeded to full-text review.

#### 2.4.4. Full-Text Review

**Figure 1:**
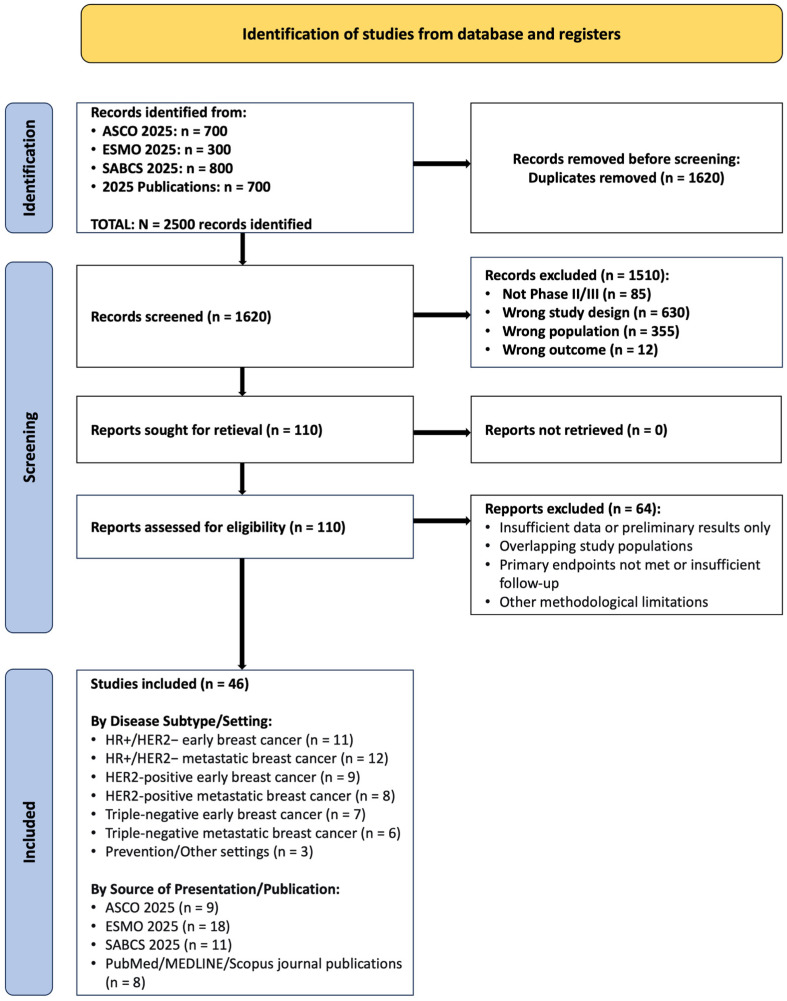
PRISMA flow diagram of the study selection process. This PRISMA flow diagram illustrates the study selection process for the systematic review. A total of 2500 records were identified from major 2025 conference proceedings (ASCO, ESMO, SABCS) and biomedical databases (PubMed/MEDLINE, Embase). After removing 1620 duplicates and screening for eligibility, 110 full-text reports were assessed. Ultimately, 50 Phase II/III clinical trials met all inclusion criteria and were incorporated into the final synthesis.

The full-text articles, conference presentation slides, and/or detailed abstract supplements for all records passing the initial screen were retrieved. Reviewers independently assessed these documents against the full eligibility criteria. Reasons for exclusion at this stage were documented for each record. The detailed results of this process, including the number of studies identified, screened, and included at each stage, are summarized and tabulated in Figure 1 (PRISMA Flow Summary). The PRISMA 2020 Checklist for Systematic Reviews is presented in the Supplementary File S1. The process was piloted on a random sample of 10 records to ensure consistent application of criteria between reviewers.

#### 2.4.5. Data Management and Synthesis

Extracted data were managed using a structured electronic database with audit trails. Given the heterogeneity in interventions, populations, and outcome measures across studies, a meta-analytic approach was not feasible. Instead, a narrative synthesis was conducted following established guidelines for systematic reviews without meta-analysis. Synthesis was organized thematically by disease stage (early vs. metastatic) and molecular subtype, with further subdivision by therapeutic class or mechanism. Within each thematic category, studies were compared and contrasted with emphasis on consistency of findings, magnitude of effects, and clinical relevance.

### 2.5. Risk of Bias Assessment Strategy and Tool

The revised Cochrane Risk of Bias tool for randomized trials (RoB 2, version 2019) was employed to assess methodological quality across five domains: (1) randomization process; (2) deviations from intended interventions; (3) missing outcome data; (4) outcome measurement; and (5) selection of reported results. Two independent reviewers assessed risk of bias for each included study, with discrepancies resolved through discussion. For conference abstracts with limited methodological details, risk of bias was assessed based on available information with appropriate notation of concerns. Studies were categorized as having “low risk,” “some concerns,” or “high risk” of bias, with these judgments informing the strength of evidence statements in the synthesis.

### 2.6. Evidence Grading and Clinical Relevance Assessment

The strength of evidence for key findings was informally graded based on study design, risk of bias, consistency across trials, precision of effect estimates, and directness of outcome measures to clinical decision-making. Clinical relevance was assessed based on magnitude of treatment effects, applicability to broad patient populations, feasibility of implementation, and impact on existing treatment paradigms. Practice-changing potential was defined as evidence likely to alter treatment guidelines, regulatory approvals, or standard care patterns in major practice settings.

## 3. Results

### 3.1. Study Selection Results

The study selection process is summarized in the PRISMA flow diagram (Figure 1). Initial searches across conference programs and scientific databases in 2025 yielded 2500 records. Following the removal of duplicates and exclusion of records that did not meet pre-defined criteria (e.g., wrong study design or population), 110 full-text articles were evaluated. Of these, 50 pivotal studies were selected for inclusion based on their status as Phase II or III randomized controlled trials reporting practice-changing efficacy or safety data in breast cancer. The final evidence base encompassed studies across all major molecular subtypes and disease stages, with a predominance of data sourced from recent presentations at ESMO (*n* = 19), SABCS (*n* = 11), and ASCO (*n* = 10) 2025, supplemented by 10 peer-reviewed journal publications.

### 3.2. Study Selection and Characteristics

The focused selection process identified 50 prospective trials that met inclusion criteria and represented the most significant advances in breast cancer therapeutics during 2025 (Figure 1). These trials comprised 34 Phase III randomized controlled trials (68%), 14 Phase II trials (28%) with practice-changing potential, one cohort prospective trial (2%) and one meta-analysis (2%). Sample sizes ranged from 50 to 4690 participants (median: 387.5). Geographically, trials represented global collaborations with significant representation from North America, Europe, and Asia.

By disease stage, 25 studies (50%) focused on early breast cancer (Stages I–III), 23 studies (46%) addressed metastatic breast cancer, and 2 studies (4%) addressed prevention. According to molecular subtype, 18 studies (36%) focused on HR+/HER2− disease, 13 studies (26%) on HER2-positive disease, 17 studies (34%) on triple-negative breast cancer, and 2 studies (4%) focused on prevention/other settings.

### 3.3. Risk of Bias Assessment

The revised Cochrane Risk of Bias tool for randomized trials (RoB 2) was applied exclusively to the 34 Phase III randomized controlled trials included in this review. Among these, 22 (65%) were judged to have low risk of bias, 8 (24%) raised some concerns (primarily related to missing outcome data in interim analyses or selective reporting in conference abstracts), and 4 (12%) were considered at high risk of bias (typically open-label studies with subjective endpoints not assessed by blinded independent review). The most frequent concerns involved lack of blinding in open-label designs (particularly relevant for patient-reported outcomes) and incomplete outcome data in interim analyses. Overall, the Phase III evidence base was judged to be of moderate to high quality, with most concerns relating to the preliminary nature of some presentations rather than fundamental methodological flaws. The remaining 16 studies (14 Phase II trials, one meta-analysis, and one prospective cohort study) were not evaluated with the RoB 2 tool, but their methodological limitations are acknowledged in the narrative synthesis.

### 3.4. Thematic Synthesis of Findings

#### 3.4.1. Early Breast Cancer: HR+/HER2− Subtype

##### Adjuvant CDK4/6 Inhibitors: Mature Survival Data Establishes New Standard

**Figure 2:**
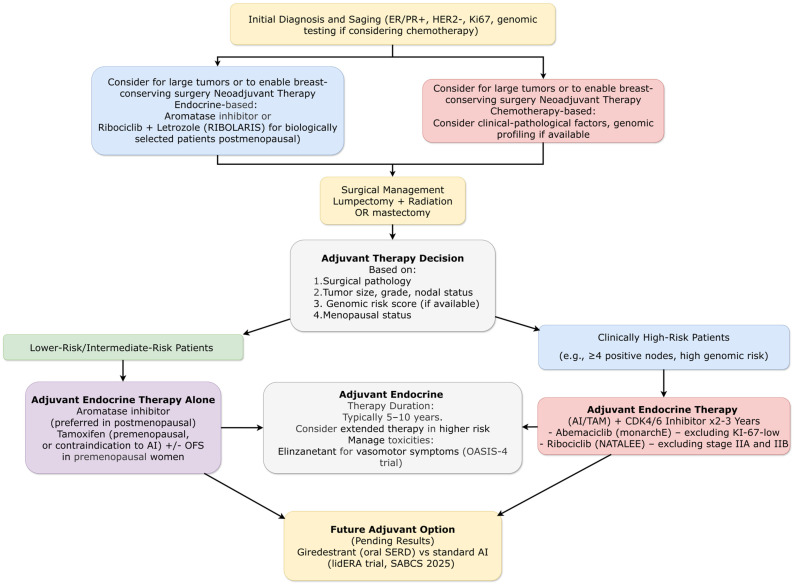
Treatment Algorithm for Early Hormone Receptor-Positive (HR+) Breast Cancer: Integrating Neoadjuvant Therapy, Surgery, and Personalized Adjuvant Management. This figure outlines a modern, personalized treatment algorithm for localized hormone receptor-positive (HR+) breast cancer. The pathway begins with neoadjuvant endocrine-based therapy, with the option of intensifying treatment with ribociclib and letrozole for biologically selected, higher-risk patients, as informed by trials such as RIBOLARIS. Following surgical management, adjuvant therapy decisions are personalized based on a synthesis of surgical pathology, traditional clinicopathological factors, genomic risk score, and menopausal status. The algorithm concludes with recommendations for the duration of adjuvant endocrine therapy and highlights the integration of supportive care, specifically the use of elinzanetant for managing treatment-associated vasomotor symptoms, to improve adherence and quality of life.

The most practice-changing advance in early HR+/HER2− breast cancer came from mature OS analyses of adjuvant cyclin-dependent kinase 4/6 (CDK4/6) inhibitor trials. The monarchE trial (ESMO 2025) with 6.3-year median follow-up demonstrated statistically significant OS benefit for adjuvant abemaciclib plus endocrine therapy versus endocrine therapy alone in high-risk, node-positive HR+/HER2− early breast cancer (HR = 0.842; 95% CI: 0.722–0.981; *p* = 0.0273). This represented the first OS benefit demonstration for any CDK4/6 inhibitor in the adjuvant setting (Figure 2). The 5-year OS rates were 86.8% versus 85% (absolute difference 1.8%), with consistent benefit across subgroups including menopausal status and prior chemotherapy. Invasive disease-free survival benefit remained durable (HR = 0.734, 95% CI 0.657–0.820), and distant relapse-free survival similarly favored abemaciclib (HR = 0.746, 95% CI 0.662–0.840). However, the DRFS benefit of abemaciclib in Cohort 1 (Ki-67 < 20%) was no longer apparent after the 4th year, highlighting the importance of selecting high-risk patients. Long-term safety data with all patients off treatment for over 4 years confirmed no new delayed toxicity signals, with the most common grade 3–4 treatment-related adverse events during the treatment period being neutropenia and diarrhea [4].

The NATALEE trial 5-year analysis (ESMO 2025) showed sustained IDFS benefit with adjuvant ribociclib plus endocrine therapy (HR = 0.716; 95% CI: 0.618–0.829), with all patients off treatment for a median of 2 years (Figure 2). Notably, significant benefit emerged in the high-risk node-negative subgroup (HR = 0.606; 95% CI: 0.372–0.986), expanding the potential population for adjuvant CDK4/6 inhibition. A positive trend for OS was observed (HR = 0.800; 95% CI: 0.637–1.003), though not yet statistically significant. The safety profile remained manageable, with no new signals identified post-treatment. The most common grade 3/4 TRAEs during treatment were neutropenia and hepatobiliary toxicity [5].

##### Biomarker-Driven De-Escalation Strategies

The phase II RIBOLARIS trial (ESMO 2025) validated a novel response-guided approach to chemotherapy de-escalation. Patients with clinically high-risk HR+/HER2− early breast cancer received neoadjuvant ribociclib plus letrozole, with postoperative treatment decisions guided by PAM50-based Risk of Recurrence (ROR) score (Figure 2). Among 686 patients, 52.6% achieved low ROR scores at surgery and thus omitted adjuvant chemotherapy. The disease progression rate during neoadjuvant therapy was minimal (2.19%; 95% CI: 1.23–3.58), meeting prespecified safety thresholds. This approach represents a paradigm shift toward biology-driven rather than clinicopathologic feature-driven treatment intensity decisions [6].

##### Fertility Preservation and Endocrine Therapy Interruption

Long-term follow-up (71 months median) from the POSITIVE trial (ESMO 2025) provided robust reassurance regarding temporary interruption of adjuvant endocrine therapy for pregnancy attempts. Compared to a matched cohort from SOFT/TEXT, treatment interruption was not associated with worse breast cancer-free interval outcomes (BCFI: 86.8 vs. 87.7, HR = 0.88; 95% CI: 0.66–1.18). Among evaluable patients, 76% achieved at least one pregnancy, with 91% of those having at least one live birth. Pregnancy complication rates and congenital anomaly incidence (1.8%) were consistent with the general population, and most patients (82%) successfully resumed endocrine therapy after the interruption period [7].

##### Next-Generation Endocrine Agents: Biological Activity Validation

Window-of-opportunity studies provided early validation for novel endocrine agents with distinct mechanisms of action:

The EMPRESS trial (ESMO 2025) evaluated giredestrant, an oral selective ER degrader, in premenopausal women with HR+/HER2− early breast cancer and Ki67 ≥ 10%. Without GnRH analogs, 15 days of preoperative giredestrant demonstrated superior Ki67 reduction compared to tamoxifen (−73% vs. −51%; *p* < 0.001) and higher complete cell cycle arrest rates (Ki67 ≤ 2.7%: 17.5% vs. 4.5%; *p* = 0.074). This challenges the paradigm that ovarian suppression is mandatory for effective endocrine therapy in premenopausal women (Figure 2) [8].

The TACTIVE-N trial (ESMO 2025) investigated vepdegestrant, an oral PROTAC ER degrader, in the neoadjuvant setting. The agent induced profound Ki67 reduction (geometric mean ratio 0.286, equivalent to −71.4% change) comparable to anastrozole, while achieving robust ER protein degradation (median reduction −94.4% at surgery). Vepdegestrant demonstrated favorable tolerability with low discontinuation rates (3% vs. 8% for anastrozole) [9].

Furthermore, the landmark Phase III lidERA trial (NCT04961996), presented at SABCS 2025, has delivered practice-changing results in the adjuvant setting. This global, randomized, open-label study compared giredestrant versus standard-of-care endocrine therapy (tamoxifen or an aromatase inhibitor) as adjuvant treatment for patients with ER+/HER2-negative, Stage I–III early breast cancer at higher risk of recurrence. With a median follow-up of 32.3 months, giredestrant demonstrated a statistically significant and clinically meaningful 30% reduction in the risk of invasive disease recurrence or death (IDFS HR = 0.70; 95% CI: 0.57–0.87; *p* = 0.0014). The 3-year IDFS rates were 92.4% versus 89.6%. A significant improvement in distant recurrence-free interval (DRFI; HR = 0.69) was also observed. Interim OS data showed a positive trend (HR = 0.79). The safety profile was favorable and consistent with the known profile of oral SERDs, with a lower treatment discontinuation rate compared to standard endocrine therapy (5.3% vs. 8.2%), largely driven by fewer discontinuations due to musculoskeletal and vasomotor symptoms. These results position giredestrant as a potential new standard of care in the adjuvant setting, with regulatory filings planned for 2026 (Figure 2) [10].

##### Long-Term Endocrine Therapy Strategies

The 15-year updates from SOFT and TEXT trials (ASCO 2025) provided mature data on ovarian function suppression strategies in premenopausal women. In the combined analysis, exemestane plus ovarian suppression continued to demonstrate superiority over tamoxifen plus ovarian suppression for breast cancer-free interval (87.6% vs. 83.7%, absolute benefit of 3.9%, HR 0.75) and distant recurrence-free interval (absolute benefit 4.7%). The benefit was most pronounced in high-risk subgroups including very young women (<35 years and/or grade 3) and those with grade 3 tumors [11,12].

##### Management of Treatment-Related Symptoms

The phase III OASIS-4 trial (ASCO 2025) addressed the critical quality-of-life issue of vasomotor symptoms in women receiving adjuvant endocrine therapy for HR+ early breast cancer. Elinzanetant, a dual neurokinin-1 and neurokinin-3 receptor antagonist, significantly reduced the frequency and severity of vasomotor symptoms compared to placebo (mean difference in daily frequency −3.5; *p* < 0.0001). This represents the first highly effective non-hormonal intervention for this common treatment-related toxicity that often impairs adherence [13].

#### 3.4.2. Early Breast Cancer: HER2-Positive Subtype

##### Adjuvant Therapy for High-Risk Residual Disease: New Standard Established

**Figure 3:**
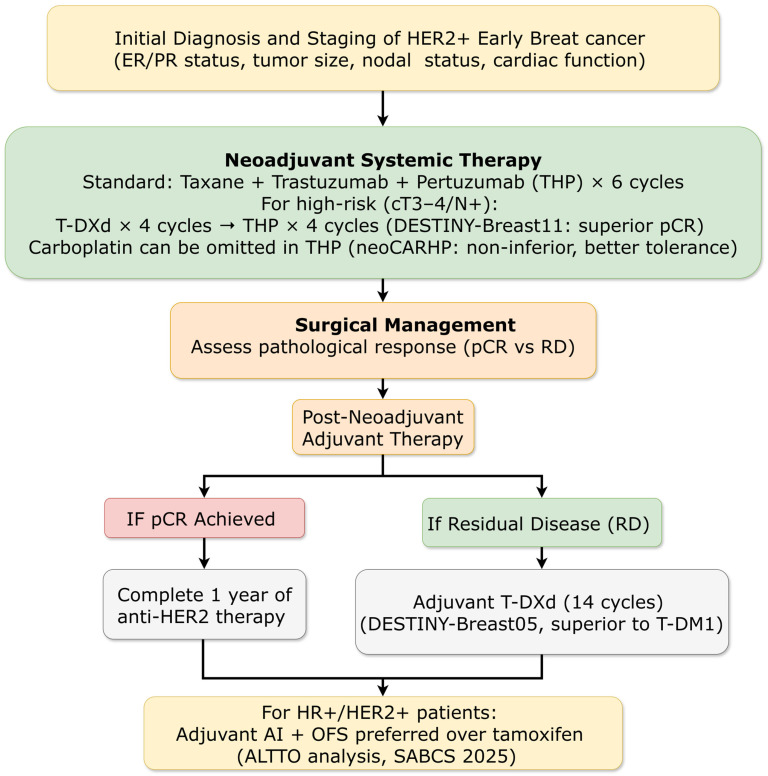
Contemporary Treatment Algorithm for Non-Metastatic HER2-Positive Breast Cancer: Risk-Adapted Neoadjuvant Strategies and Response-Guided Adjuvant Therapy. This figure presents a contemporary treatment algorithm for non-metastatic HER2-positive breast cancer, emphasizing risk-adapted and response-guided strategies. Following initial diagnosis and staging, neoadjuvant systemic therapy is tailored: standard-risk cases receive THP, while high-risk patients (cT3-4/N+) are offered trastuzumab deruxtecan (T-DXd) followed by THP, based on the superior pCR rates demonstrated in the DESTINY-Breast11 trial, with the option to omit carboplatin from the backbone for better tolerability (neoCARHP trial). After surgical assessment of response, adjuvant therapy is personalized: patients achieving a pCR complete one year of anti-HER2 therapy, whereas those with residual disease receive adjuvant T-DXd, which has proven superior to trastuzumab emtansine (T-DM1) in the DESTINY-Breast05 trial. For patients with co-positive hormone receptors, adjuvant aromatase inhibitor with ovarian function suppression is recommended over tamoxifen, based on evidence from the ALTTO analysis.

The phase III DESTINY-Breast05 trial (ESMO 2025) compared adjuvant trastuzumab deruxtecan (T-DXd) to trastuzumab emtansine (T-DM1) in patients with HER2-positive early breast cancer who had residual invasive disease after neoadjuvant therapy (inoperable at diagnosis or residual node-positive) (Figure 3). T-DXd demonstrated a dramatic 53% reduction in the risk of invasive recurrence or death (IDFS HR = 0.47; 95% CI: 0.34–0.66; *p* < 0.0001), with 3-year IDFS rates of 92.4% versus 83.7%. The benefit was consistent across all prespecified subgroups, including those with the poorest prognoses: patients with inoperable disease at presentation, hormone receptor-negative disease, and those with extensive nodal involvement (≥4 positive nodes). Notably, a numerical reduction in central nervous system recurrences was observed (2.1% vs. 3.8%), suggesting T-DXd’s potent payload may confer protection against this challenging metastatic site. However, vigilant monitoring for interstitial lung disease (ILD) remains crucial, with adjudicated drug-related ILD incidence of 9.6% (any grade) in this adjuvant study, including 0.9% grade ≥ 3 and 0.2% fatal events [14].

##### Neoadjuvant Strategies: Chemotherapy De-Escalation and ADC Integration

The phase III DESTINY-Breast11 trial (ESMO 2025) evaluated T-DXd in the neoadjuvant setting for patients with high-risk, previously untreated HER2-positive early breast cancer (Figure 3). The investigational arm of T-DXd for four cycles followed by four cycles of taxane, trastuzumab, and pertuzumab (THP) significantly improved pathological complete response rates compared to standard anthracycline-based therapy (dose-dense doxorubicin/cyclophosphamide followed by THP): 67.3% versus 56.3% (Δ11.2%; *p* = 0.003). Benefit was consistent across subgroups, with particularly impressive improvement in hormone receptor-negative patients (pCR increase 16.1%). This trial provided the first phase III evidence that replacing a component of traditional chemotherapy with an antibody-drug conjugate can improve efficacy while reducing toxicity. The T-DXd to THP sequence demonstrated a more favorable safety profile than anthracycline-based therapy, with lower rates of grade ≥ 3 adverse events (37.5% vs. 55.8%), serious adverse events, and left ventricular dysfunction (1.3% vs. 6.1%) [15].

Complementary data from the phase III neoCARHP trial (ASCO 2025) addressed chemotherapy de-escalation within standard dual HER2-blockade regimens. Omitting carboplatin from docetaxel plus trastuzumab and pertuzumab was non-inferior in terms of pCR (64.1% vs. 65.9%) while significantly improving tolerability, with reduced hematological toxicity (grade 3/4 neutropenia: 6.8% vs. 16.4%) and gastrointestinal side effects [16].

##### Novel HER2-Targeted Agents in Early Disease

The phase II TQB2102 trial evaluated a novel bispecific antibody-drug conjugate in HER2-positive Stage II–III breast cancer in the neoadjuvant setting. Across multiple cohorts, total pathological complete response rates ranged from 57.7% to 76.9%, all exceeding the prespecified 40% threshold for activity. The agent demonstrated manageable toxicity, with grade ≥ 3 treatment-related adverse events occurring in 23.1–30.8% of patients and no treatment-related deaths [17].

Similarly, the phase II SHR-A1811 ± Pyrotinib trial investigated another novel ADC with or without pyrotinib in Stage II–III HER2-positive breast cancer. Pathological complete response rates were 63.2% with monotherapy, 62.5% with combination therapy, and 64.4% with standard paclitaxel/carboplatin plus dual HER2-blockade, demonstrating robust ADC activity comparable to chemotherapy-based regimens [18].

##### Optimizing Adjuvant Endocrine Therapy in HER2+/HR+ Disease

The SABCS 2025 presentation of an exploratory analysis from the ALTTO trial provided pivotal evidence for tailoring adjuvant endocrine therapy in HER2+/HR+ early breast cancer. With 9.9 years median follow-up, aromatase inhibitor (AI)-based therapy (with ovarian function suppression [OFS] for premenopausal patients) was associated with a significant 35% reduction in the risk of disease recurrence or death compared to tamoxifen (SERM) alone (adjusted HR = 0.65). The benefit was most striking in premenopausal patients receiving AI + OFS versus tamoxifen alone (HR = 0.44; 10-year DFS 90.0% vs. 77.6%). This analysis strongly supports AI + OFS as the preferred adjuvant endocrine strategy over tamoxifen-based regimens in HER2+/HR+ early breast cancer to maximize dual-pathway blockade alongside anti-HER2 therapy [19].

#### 3.4.3. Early Breast Cancer: Triple-Negative Subtype

##### Long-Term Immunotherapy Benefits Confirmed

**Figure 4:**
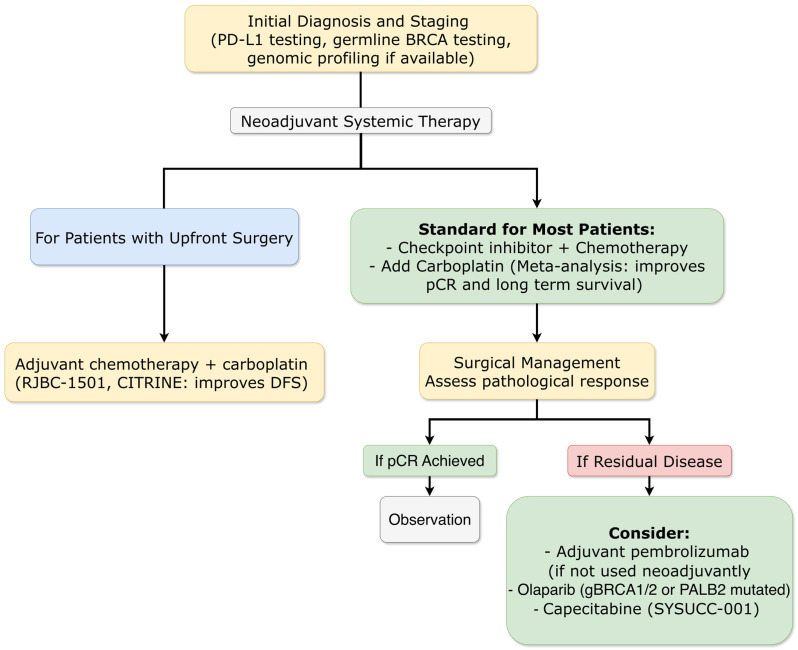
Biomarker-Driven Treatment Algorithm for Non-Metastatic Triple-Negative Breast Cancer (TNBC): From Initial Testing to Response-Adapted Adjuvant Strategies. This figure outlines a contemporary treatment algorithm for non-metastatic triple-negative breast cancer (TNBC), anchored in biomarker testing and response-adapted therapy. Initial management mandates PD-L1 and germline BRCA (gBRCA) testing to guide systemic treatment. For most patients, the recommended neoadjuvant regimen combines a checkpoint inhibitor with chemotherapy, with the addition of carboplatin supported by meta-analyses demonstrating improved pCR rates and long-term survival. Following surgery, patients achieving a pCR typically proceed to observation. For those with residual disease, adjuvant therapy is biomarker-directed: olaparib is recommended for carriers of a gBRCA mutation (and potentially PALB2), while extended adjuvant capecitabine represents an alternative, chemotherapy-based strategy to reduce recurrence risk.

The 7-year follow-up analysis of the GeparNuevo trial (ESMO 2025) provided mature evidence regarding the durability of immunotherapy benefit in early triple-negative breast cancer. Adding durvalumab to neoadjuvant chemotherapy (without adjuvant continuation) resulted in sustained improvements in IDFS (HR = 0.56; 95% CI: 0.32–0.99) and OS (HR = 0.33; 95% CI: 0.14–0.79). Notably, the survival benefit was observed irrespective of pathological complete response status, with non-pCR patients showing particularly pronounced OS improvement (HR = 0.29). These data challenge the paradigm that immunotherapy benefit is restricted to pCR achievers (Figure 4) [20].

Complementary mature evidence from the phase III KEYNOTE-522 trial further solidified the role of immunotherapy in early TNBC. A 2025 correspondence questioned the consistency of pCR benefit across trial enrollment periods, noting that the pCR improvement with pembrolizumab was 13.6% in the first 602 patients but only 0.8% in the subsequent 572 patients [21].

Despite this attenuation in pCR benefit, the updated efficacy analysis presented at SABCS 2025 confirmed that patients enrolled in the second half of the trial experienced similar improvements in event-free survival and overall survival as the overall population, underscoring the durability of survival benefit regardless of pCR magnitude [22].

##### Biomarker Refinement for Patient Selection

Translational analysis from the phase III (ESMO 2025) identified tumor-infiltrating lymphocytes (TILs) as a critical predictive biomarker for benefit from adjuvant avelumab in high-risk triple-negative breast cancer. Patients with high baseline TILs (≥30%) derived significant benefit, with 69% reduction in distant recurrence risk (HR = 0.31; 95% CI: 0.11–0.87). In the non-pCR population, this benefit was even more pronounced (HR = 0.20). Interestingly, TILs in residual disease showed stronger prognostic value than baseline TILs. These findings suggest TIL assessment could refine patient selection for adjuvant immunotherapy beyond traditional metrics like pCR or PD-L1 status [23].

##### Global Access Strategies

The phase II PLANET trial (ESMO 2025) addressed the critical issue of immunotherapy accessibility in resource-limited settings. Adding ultra-low-dose pembrolizumab (50 mg every 6 weeks, for three doses total) to neoadjuvant anthracycline/taxane chemotherapy significantly improved pCR rates compared to chemotherapy alone (53.8% vs. 40.5%; one-sided *p* = 0.047), with an absolute increase of 13.3%. This cost-effective strategy (approximately 90% cost reduction compared to standard dosing) offers a viable model for expanding global access to immunotherapy while maintaining meaningful efficacy [24].

##### Chemotherapy Backbone Optimization: The Definitive Role of Platinum Agents

The phase III NRG-BR003 trial (ASCO 2025) helped define the limits of chemotherapy intensification in modern triple-negative breast cancer management. Adding carboplatin to dose-dense doxorubicin/cyclophosphamide followed by weekly paclitaxel did not improve IDFS in node-positive or high-risk node-negative disease (5-year IDFS 82.7% vs. 77.8%; HR = 0.78; *p* = 0.12). This suggests that in the era of effective immunotherapy, additional chemotherapy intensification provides diminishing returns while increasing toxicity [25].

However, SABCS 2025 provided conclusive evidence supporting carboplatin’s role in specific contexts. A pooled meta-analysis of BrightTNess, CALGB 40603, and GeparSixto trials (Felsheim et al.) confirmed that the addition of neoadjuvant carboplatin (16.1% absolute pCR increase) translates into a significant long-term survival benefit, with a 7% absolute improvement in 5-year Event-Free Survival (EFS; HR = 0.70), independent of BRCA status [26]. Furthermore, two large Chinese Phase 3 adjuvant trials, RJBC-1501 (Chen et al.) and CITRINE (Liu et al.), demonstrated that adding carboplatin to taxane-based adjuvant chemotherapy significantly improves disease-free survival (DFS; HR = 0.66 and HR = 0.64, respectively) in patients with stage I–III TNBC undergoing upfront surgery, reducing the risk of early recurrence and distant metastasis [27,28]. This body of evidence solidifies carboplatin as a standard component of chemotherapy for high-risk early-stage TNBC (Figure 4).

##### Novel Chemotherapy-Free Neoadjuvant Approaches

SABCS 2025 featured innovative strategies to de-escalate chemotherapy in genetically selected populations. The Phase 2 TBCRC-056 trial (Mayer et al.) evaluated the chemotherapy-free neoadjuvant combination of the poly(ADP-ribose) polymerase (PARP) inhibitor niraparib and the anti-programmed cell death protein 1 (PD-1) dostarlimab in patients with germline BRCA1/2 or PALB2 mutated, HER2-negative early breast cancer [29]. In the TNBC cohort, a pCR rate of 50% was achieved, providing proof-of-concept for effective chemotherapy-free regimens in this molecular subset. The ongoing OlympiaN trial (Tung et al.) testing olaparib ± durvalumab in a similar population was also highlighted, underscoring the move towards personalized, biology-driven neoadjuvant strategies [30].

##### Novel ADC-Based Neoadjuvant Approaches

The phase II NeoSTAR trial (ASCO 2025) evaluated sacituzumab govitecan plus pembrolizumab as neoadjuvant therapy for early triple-negative breast cancer. The pCR rate after four cycles was 32%, increasing to 50% when including patients who received additional non-anthracycline chemotherapy [31]. While these rates are modest compared to chemotherapy-based regimens, the trial demonstrated the feasibility of chemotherapy-free neoadjuvant approaches and identified potential biomarkers of response, including BRCA mutation status (pCR rate 60% in BRCA carriers).

##### Extended Adjuvant Strategies

The phase III SYSUCC-001 trial provided 10-year follow-up data on metronomic capecitabine as extended adjuvant therapy for early triple-negative breast cancer. The 10-year DFS was 78.1% with capecitabine versus 66.6% with observation (HR = 0.61), with particular benefit observed in FOXC1-high tumors. These data support extended adjuvant capecitabine as a cost-effective strategy for reducing recurrence risk in early TNBC (Figure 4) [32].

#### 3.4.4. Metastatic Breast Cancer: HER2-Positive Subtype

##### First-Line Therapy Redefined

**Figure 5:**
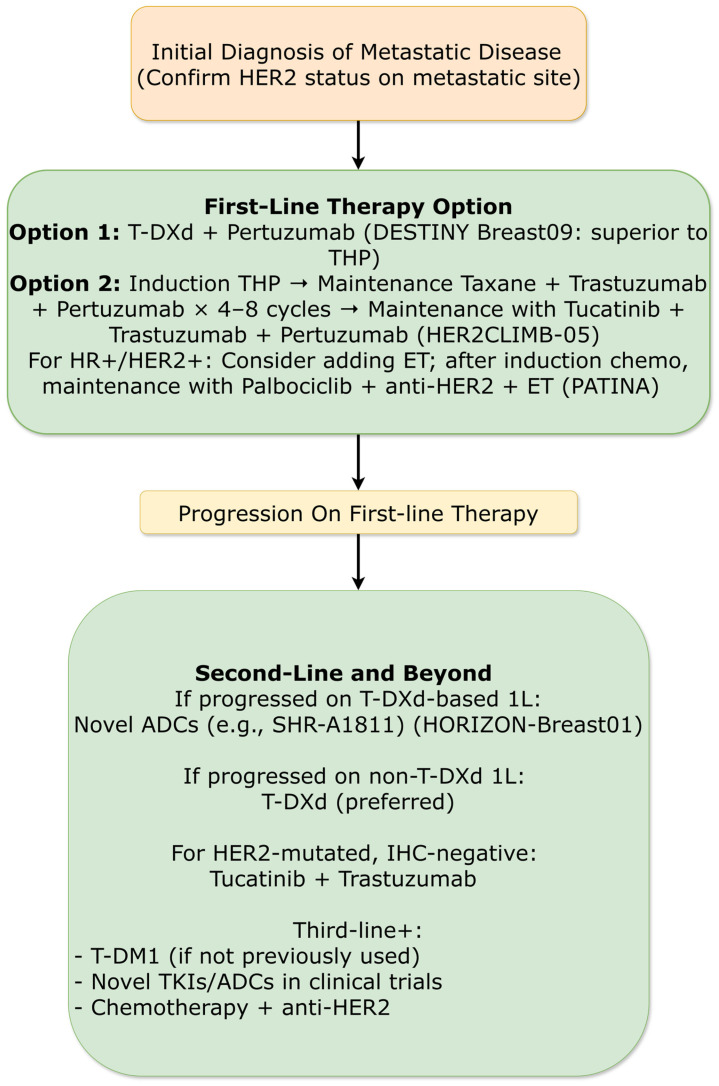
Treatment Algorithm for Metastatic HER2-Positive Breast Cancer: Sequential Therapy from First-Line to Later Lines Based on Biomarkers and Prior Treatments. This figure outlines a modern, biomarker-guided sequential treatment strategy for metastatic HER2-positive breast cancer. The algorithm begins with confirmation of HER2 status on metastatic tissue and presents two evidence-based first-line options: trastuzumab deruxtecan (T-DXd) plus pertuzumab (per DESTINY-Breast09) or induction chemotherapy with dual HER2-blockade followed by maintenance therapy incorporating the brain-penetrant TKI tucatinib (HER2CLIMB-05). For patients with co-positive hormone receptors, endocrine therapy integration is recommended, with data supporting maintenance palbociclib after induction (PATINA). Upon disease progression, second-line therapy is personalized: patients progressing on a T-DXd-based first line may receive novel ADCs (e.g., SHR-A1811, HORIZON-Breast01), while those progressing on a non-T-DXd regimen typically receive T-DXd. A distinct pathway is defined for HER2-mutated, immunohistochemistry-negative tumors (tucatinib + trastuzumab). The algorithm emphasizes a structured sequence of effective therapies, reserving later-line options like T-DM1, novel agents in trials, or chemotherapy combinations.

The phase III DESTINY-Breast09 trial (ASCO/ESMO 2025) compared trastuzumab deruxtecan plus pertuzumab to the previous standard THP in previously untreated HER2-positive metastatic breast cancer. T-DXd plus pertuzumab demonstrated superior PFS (median 40.7 vs. 26.9 months; HR = 0.58; 95% CI: 0.44–0.71; *p* < 0.00001), effectively redefining frontline treatment expectations (Figure 5). The benefit was consistent across subgroups, including those with PIK3CA mutations, where T-DXd plus pertuzumab more than doubled median PFS compared to THP (25.3 vs. 10.9 months; HR = 0.46). Objective response rates were significantly higher with T-DXd combination (89.9% vs. 80.3%), with complete response rates of 28.4% versus 15.2%. Interim OS data showed a positive trend (HR = 0.74; 95% CI: 0.54–1.01), though still immature. Safety profiles were manageable, with T-DXd plus pertuzumab showing higher rates of nausea and hematological toxicity but lower rates of alopecia and neuropathy compared to THP. Adjudicated drug-related interstitial lung disease occurred in 12.0% of T-DXd patients (grade ≥ 3: 3.2%) [33,34].

Concurrently, SABCS 2025 featured pivotal data from the HER2CLIMB-05 trial (Hamilton et al.), which established a new maintenance standard (Figure 5). Adding the brain-penetrant tyrosine kinase inhibitor (TKI) tucatinib to trastuzumab + pertuzumab (HP) maintenance after induction with THP significantly improved PFS compared to placebo + HP (median 24.9 vs. 16.3 months; HR = 0.58). This provides a potent strategy to extend disease control post-induction, particularly relevant for patients at risk of central nervous system (CNS) progression. Furthermore, patient-reported outcomes from DESTINY-Breast09 (Rimović et al.) presented at SABCS indicated that despite different toxicity profiles, patients reported similar overall levels of being bothered by treatment side effects with T-DXd+P versus THP, supporting the tolerability of this chemotherapy-sparing frontline option [35].

##### Next-Generation ADCs in Later Lines

The phase III HORIZON-Breast01 trial (ESMO 2025) established the novel HER2-targeting antibody-drug conjugate SHR-A1811 as a superior option compared to pyrotinib plus capecitabine in patients with HER2-positive metastatic breast cancer previously treated with trastuzumab and a taxane (Figure 5) [36]. SHR-A1811 demonstrated remarkable efficacy: median PFS 30.6 vs. 8.3 months (HR = 0.22; 95% CI: 0.15–0.34; *p* < 0.0001) and confirmed objective response rate 81.7% vs. 55.9%. The safety profile was distinct from T-DXd, with lower rates of interstitial lung disease (2.8% any grade, 0.7% grade ≥ 3) and different toxicity patterns dominated by hematological events rather than gastrointestinal side effects [37].

##### Maintenance Strategies for HR+/HER2+ Disease

The phase III PATINA trial (ESMO 2025) validated a novel maintenance approach for patients with HR+/HER2+ metastatic breast cancer (Figure 5). After induction chemotherapy with trastuzumab and pertuzumab (with or without taxane), patients were randomized to continue anti-HER2 therapy plus endocrine therapy with or without palbociclib. Adding palbociclib significantly improved PFS (median 44.3 vs. 29.1 months; HR = 0.74; 95% CI: 0.58–0.94; *p* = 0.0109) while preserving health-related quality of life, as confirmed by comprehensive patient-reported outcomes. This strategy effectively delays chemotherapy re-introduction and represents a more targeted approach for this dual-positive population [38].

##### HER2-Low and HER2-Mutated Populations

The phase III DESTINY-Breast04 trial published long-term survival analysis in 2025, confirming durable OS benefit with trastuzumab deruxtecan in HER2-low metastatic breast cancer (median OS 22.9 vs. 16.8 months; HR = 0.69). This established T-DXd as standard therapy for this population after 1–2 prior chemotherapies [39].

For HER2-mutated, IHC-negative disease, the phase II basket SGNTUC-019 trial demonstrated activity of tucatinib plus trastuzumab (±fulvestrant if HR+), with objective response rate of 41.9% and median PFS of 9.5 months, providing a targeted option for this molecularly defined population [40].

##### Novel Combination Approaches

The Ib/II trial of zanidatamab plus docetaxel in first-line HER2-positive advanced breast cancer showed impressive activity, with confirmed objective response rate of 90.9%, median PFS of 22.1 months, and median OS of 36.9 months. This bispecific antibody targeting two distinct HER2 epitopes represents a promising new approach in HER2-positive disease [41].

#### 3.4.5. Metastatic Breast Cancer: Triple-Negative Subtype

##### First-Line Therapy Evolution

**Figure 6:**
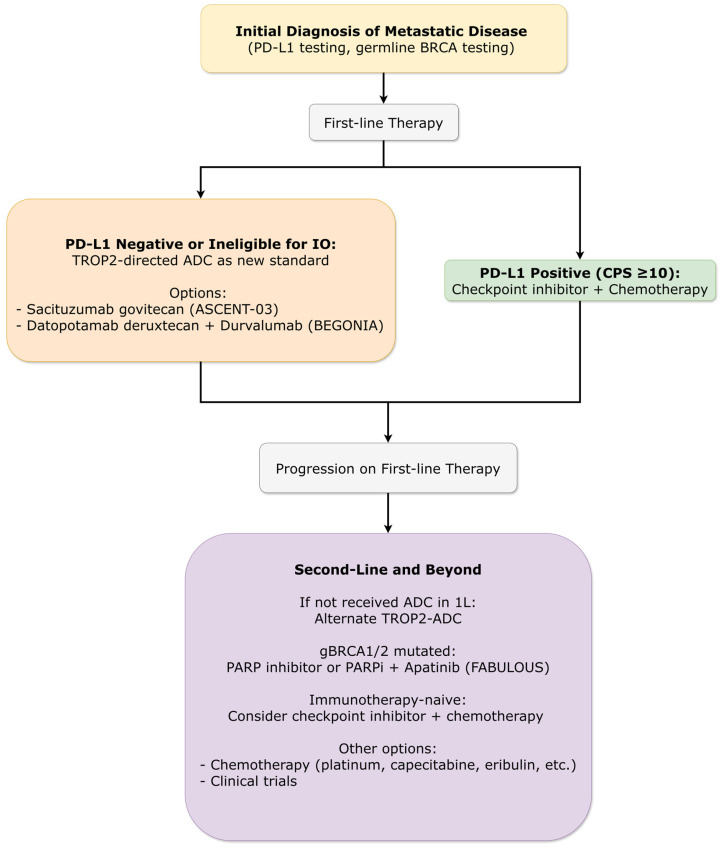
Treatment Algorithm for Metastatic Triple-Negative Breast Cancer (TNBC): Biomarker-Driven First-Line Therapy and Sequencing Strategies. This figure outlines a contemporary, biomarker-guided treatment algorithm for metastatic triple-negative breast cancer (TNBC). Initial management is determined by PD-L1 status and germline BRCA (gBRCA) mutation testing. For patients eligible for immunotherapy, first-line therapy typically consists of a PD-(L)1 inhibitor combined with chemotherapy. For those ineligible, TROP2-directed ADCs such as sacituzumab govitecan or datopotamab deruxtecan represent new first-line standards (ASCENT-03, BEGONIA trial data). Following progression on first-line therapy, treatment sequencing is tailored based on prior regimens and biomarker status, including options such as PARP inhibitors for gBRCA mutation carriers, other novel ADCs (e.g., sacituzumab tirumotecan), and subsequent lines of chemotherapy. The algorithm emphasizes the critical role of biomarker assessment and the integration of novel ADCs across the treatment continuum.

The treatment landscape for first-line metastatic triple-negative breast cancer evolved significantly in 2025, with two parallel advancements (Figure 6):

The phase III ASCENT-03 trial (ESMO 2025) established sacituzumab govitecan as superior to chemotherapy for patients ineligible for PD-(L)1 inhibitors. Median PFS was 9.7 vs. 6.9 months (HR = 0.62; 95% CI: 0.50–0.77; *p* < 0.0001), with similar objective response rates (~48%) but substantially longer duration of response (median 12.2 vs. 7.2 months). The safety profile was consistent with known SG toxicities (neutropenia, diarrhea), with lower treatment discontinuation rates than chemotherapy (4% vs. 12%) [42,43].

The phase Ib/II BEGONIA trial (ESMO 2025) demonstrated impressive activity for datopotamab deruxtecan plus durvalumab in an all-comer population regardless of PD-L1 status. In Arm 7 (any PD-L1), confirmed objective response rate was 79.0% with median PFS of 14.0 months. The combination was generally well-tolerated, with stomatitis as the most common adverse event and low rates of interstitial lung disease (no grade ≥ 3 events) [44].

As ADC use expands, managing their unique toxicities is paramount. SABCS 2025 featured detailed management guidelines for toxicities associated with the trophoblast cell-surface antigen 2 (TROP2)-directed ADC datopotamab deruxtecan (Dato-DXd), based on experience from trials like TROPION-Breast01 [45]. A dedicated presentation provided protocols for managing ocular toxicity (keratitis, dry eye; occurring in 47% of patients) and stomatitis/mucositis (occurring in 60% of patients), emphasizing proactive monitoring, early intervention, and structured dose modifications to mitigate these effects and maintain treatment continuity.

##### Immunotherapy Combinations Refined

Primary results from ASCENT-04/KEYNOTE-D19 (ASCO 2025) suggested potential superiority of sacituzumab govitecan plus pembrolizumab over chemotherapy plus pembrolizumab in PD-L1-positive metastatic triple-negative breast cancer, with median PFS 11.2 vs. 7.8 months (HR = 0.65; *p* < 0.001). The incidence of treatment discontinuation was higher in chemo-immunotherapy arm (31% vs. 12%). However, the magnitude of benefit was modest, raising questions about the additive value of immunotherapy to highly effective ADCs and highlighting the need for predictive biomarkers to identify patients most likely to benefit from combination approaches [46].

##### Later-Line Options and Novel Targets

The phase III OptiTROP-Breast01 trial evaluated another TROP2-directed ADC, sacituzumab tirumotecan, in heavily pretreated metastatic TNBC (Figure 6). The agent demonstrated significant improvement in PFS compared to chemotherapy (median 6.7 vs. 2.5 months; HR = 0.32, *p* < 0.00001), with improved OS, providing another option in the later-line setting [47].

For patients with germline BRCA1/2 mutations, the phase III FABULOUS trial showed promising activity for fuzuloparib with or without apatinib compared to chemotherapy, with median PFS of 11.0 months for the combination versus 3.0 months for chemotherapy (HR = 0.27) [48].

#### 3.4.6. Metastatic Breast Cancer: HR+/HER2− Subtype

##### CDK4/6 Inhibitor Sequencing: De-Escalation Validated

**Figure 7:**
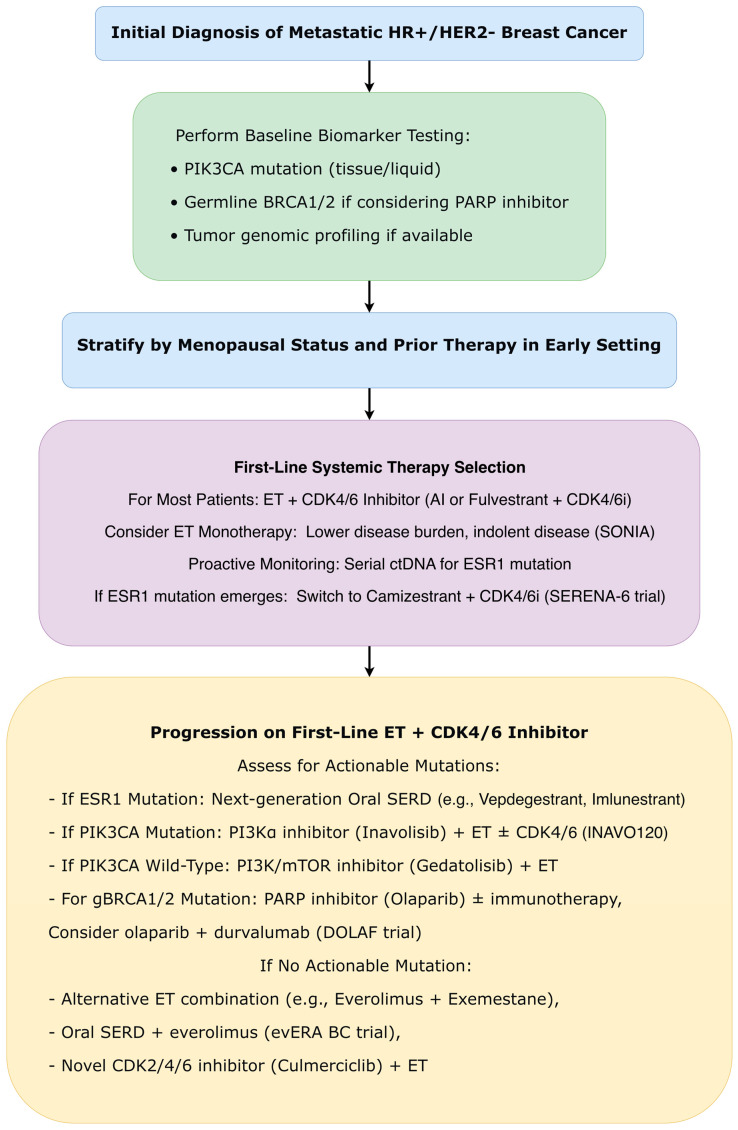
Treatment Algorithm for Metastatic Hormone Receptor-Positive (HR+), HER2-Negative Breast Cancer: A Precision Medicine Approach from First-Line to Progression. This figure presents a modern, biomarker-driven treatment algorithm for metastatic HR+/HER2− BC, reflecting the shift towards personalized, dynamic management. The pathway begins with mandatory baseline biomarker testing (ESR1, PIK3CA, gBRCA) to guide initial and subsequent therapy choices. First-line treatment is stratified: most patients receive an endocrine therapy (ET) backbone combined with a CDK4/6 inhibitor, while ET monotherapy is a valid de-escalation option for selected patients with lower disease burden, supported by the SONIA trial. A pivotal innovation is the integration of proactive monitoring via serial circulating tumor DNA (ctDNA) to detect emergent ESR1 mutations, enabling a pre-emptive therapy switch (e.g., to camizestrant) before radiographic progression, as validated in the SERENA-6 trial. Upon progression on first-line ET + CDK4/6 inhibitor, treatment selection is dictated by the identified resistance mechanism. The algorithm branches based on specific actionable mutations: next-generation oral SERDs (vepdegestrant, imlunestrant) for *ESR1* mutations, PI3Kα inhibitors (inavolisib) for PIK3CA mutations, dual PI3K/mTOR inhibitors (gedatolisib) for PIK3CA wild-type disease, and PARP inhibitors for gBRCA mutations. For tumors without a clear driver mutation, alternative endocrine-based combinations or novel agents like the CDK2/4/6 inhibitor culmerciclib are recommended. This algorithm encapsulates the contemporary paradigm of using continuous biomarker assessment to navigate a growing arsenal of targeted therapies, optimizing sequencing for improved outcomes.

The mature follow-up of the phase III SONIA trial (ESMO 2025) provided crucial level I evidence regarding optimal sequencing of CDK4/6 inhibitors. With median follow-up of 58.8 months, first-line use of a CDK4/6 inhibitor plus endocrine therapy did not improve OS compared to second-line use after progression on endocrine therapy alone (median OS 47.9 vs. 48.1 months; HR = 0.91; 95% CI: 0.77–1.07; *p* = 0.24). First-line use was associated with 74% more grade 3–4 toxicity and significantly higher drug costs. A post hoc analysis suggested potential OS benefit for first-line use in premenopausal patients (HR = 0.53) but not in postmenopausal patients (HR = 1.00). These findings support consideration of endocrine monotherapy as initial treatment for selected patients, reserving CDK4/6 inhibitors for later lines to delay cumulative toxicity while maintaining OS benefit (Figure 7) [49].

##### Novel CDK Inhibition Strategies

The phase III CULMINATE-2 trial (ESMO 2025) evaluated culmerciclib, a novel oral CDK2/4/6 inhibitor, combined with fulvestrant in the first-line setting for HR+/HER2− advanced breast cancer. The combination significantly improved investigator-assessed PFS compared to placebo plus fulvestrant (median not reached vs. 20.2 months; HR = 0.56; 95% CI: 0.40–0.78; *p* = 0.0004). The benefit was consistent across subgroups, including patients with visceral metastases (HR = 0.57) and liver metastases (HR = 0.42). Culmerciclib demonstrated a potentially improved therapeutic index compared to first-generation CDK4/6 inhibitors, with lower incidence of grade ≥ 3 neutropenia (20.3%) and low discontinuation rates due to adverse events (3.5%) [50].

##### Proactive Biomarker-Guided Strategies

The paradigm-shifting SERENA-6 trial (ASCO/ESMO 2025) introduced proactive management based on liquid biopsy monitoring (Figure 7). Patients with HR+/HER2− advanced breast cancer receiving first-line aromatase inhibitor plus CDK4/6 inhibitor underwent serial circulating tumor DNA testing every 2–3 months. Those with emergent ESR1 mutations detected before radiological progression were randomized to switch to camizestrant (next-generation oral SERD) plus CDK4/6 inhibitor or continue their original therapy. Switching to camizestrant significantly improved PFS (median 16.0 vs. 9.2 months; HR = 0.44; 95% CI: 0.31–0.60; *p* < 0.0001) [49]. Patient-reported outcomes demonstrated that camizestrant delayed time to deterioration in global health status/quality of life (HR = 0.54), pain (HR = 0.57), and physical functioning (HR = 0.74). This represents a fundamental shift from reactive to pre-emptive precision medicine [51,52].

##### Next-Generation Endocrine Agents

Multiple novel endocrine agents demonstrated efficacy in advanced settings (Figure 7):

The phase III VERITAC-2 trial (ESMO 2025) established the oral PROTAC ER degrader vepdegestrant as superior to fulvestrant in patients with ESR1-mutated ER+/HER2− advanced breast cancer previously treated with endocrine therapy and a CDK4/6 inhibitor. Vepdegestrant significantly improved PFS while demonstrating favorable patient-reported outcomes, significantly delaying time to definitive deterioration in overall health status/quality of life (HR = 0.60) and multiple functioning domains [53,54].

The phase III evERA BC trial (ESMO 2025) validated giredestrant (oral SERD) plus everolimus as an effective, all-oral option after CDK4/6 inhibitor progression, particularly in ESR1-mutant tumors (PFS HR = 0.38; *p* < 0.0001). The combination offered a chemotherapy-free alternative with manageable safety profile [55].

At SABCS 2025, updated results with 14 additional months of follow-up from the phase 3 EMBER-3 trial (Jhaveri et al.) further solidified the role of the oral SERD imlunestrant. In patients with ESR1 mutations (ESR1m), imlunestrant monotherapy showed a clinically meaningful numerical improvement in OS versus standard-of-care endocrine therapy (fulvestrant/exemestane), with an 11.4-month difference in median OS (HR = 0.60; *p* = 0.0043), though the prespecified statistical boundary was not crossed. The significant PFS benefit was sustained (HR = 0.62), and time to chemotherapy was delayed by 5.4 months. In the overall population, the combination of imlunestrant + abemaciclib significantly improved PFS versus imlunestrant monotherapy (median 10.9 vs. 5.5 months; HR = 0.58), with an early favorable OS trend (HR = 0.82). The combination exhibited a predictable safety profile and a low discontinuation rate (6%). These data support imlunestrant as an effective, all-oral option, both as monotherapy for ESR1m patients and in combination with abemaciclib for a broader population [56].

##### PI3K/AKT/mTOR Pathway Inhibition Refined

Advances in targeting this pathway included (Figure 7):

The INAVO120 final analysis (ASCO 2025) confirmed OS benefit with inavolisib (selective PI3Kα inhibitor) plus palbociclib and fulvestrant in PIK3CA-mutated, endocrine-resistant advanced breast cancer (OS HR = 0.67; 95% CI: 0.48–0.94; *p* = 0.019). Median OS was 34.0 vs. 27.0 months, with substantially longer time to subsequent therapy (35.6 vs. 12.6 months; HR = 0.43). Overall, adverse events were manageable in the inavolisib arm; however, the incidence of hyperglycemia, stomatitis, diarrhea, and ocular toxicities was higher than in the placebo group [57].

The phase III VIKTORIA-1 trial (ESMO 2025 and SABCS 2025) validated dual PI3K/mTOR inhibition with gedatolisib in PIK3CA wild-type disease after CDK4/6 inhibitor progression. Gedatolisib plus fulvestrant, with or without palbociclib, demonstrated significant PFS improvement versus fulvestrant alone (triplet: 9.3 vs. 2.0 months; HR, 0.24; 95% CI, 0.17–0.35; doublet: 7.4 vs. 2.0 months; HR, 0.33; 95% CI, 0.24–0.48; both *p* < 0.0001) [58].

##### Novel Resistance Mechanisms and Targeting Strategies

Translational analyses from multiple trials identified emerging resistance mechanisms to CDK4/6 inhibitors, including RB1 loss, FAT1 mutations, and CCNE1 amplification. Early-phase trials evaluated novel agents targeting these pathways, including AURORA kinase inhibitors for CCNE1-amplified tumors and novel CDK2-selective inhibitors [59,60].

#### 3.4.7. Prevention and Special Populations

##### Prevention in High-Risk Populations

The phase III LIBER trial evaluated letrozole for breast cancer prevention in postmenopausal women with germline BRCA1/2 mutations. While the trial showed a non-significant trend favoring letrozole (HR = 0.70, *p* = 0.416), it demonstrated the feasibility of prevention strategies in genetically high-risk populations and provided insights into endocrine prevention in BRCA carriers [61].

The phase III Tam-01 trial showed that low-dose tamoxifen (5 mg/day) significantly reduced breast events in women with breast intraepithelial neoplasia (HR = 0.58), providing a lower-toxicity option for risk reduction [62].

##### Special Therapeutic Scenarios

The phase II DOLAF trial evaluated durvalumab plus olaparib plus fulvestrant in ER+/HER2− metastatic breast cancer with genomic alterations (including gBRCA mutations), showing promising activity with 24-week PFS rate of 66.7% in the intent-to-treat (ITT) population and 76.3% in gBRCAm patients [63].

### 3.5. Summary of Evidence Table

Table 2 provides a comprehensive overview of all 50 included studies, summarizing key characteristics including breast cancer subtype, disease setting, study design, interventions, primary endpoints, and safety findings. This table serves as a quick reference for clinicians and researchers seeking to understand the breadth of evidence presented in 2025.

## 4. Discussion

### 4.1. Integration of Major Advances by Disease Area

The year 2025 evidence base has fundamentally reshaped breast cancer management across all subtypes and stages, with several key integrative themes emerging from the collective trial results.

For HR+/HER2− early breast cancer, the maturation of adjuvant CDK4/6 inhibitor data with proven OS benefit represents a watershed moment (Figure 2). The monarchE OS benefit (HR = 0.842) establishes abemaciclib as a new standard for high-risk node-positive disease, while NATALEE’s expansion of benefit to node-negative patients (HR = 0.606 in high-risk N0) potentially broadens the eligible population. These advances create new decision-points regarding risk stratification, treatment duration, and management of long-term toxicities. Concurrently, the validation of biomarker-driven chemotherapy de-escalation (RIBOLARIS) and reassurance regarding treatment interruption for fertility (POSITIVE) provide balancing strategies for personalizing care and addressing quality-of-life concerns. The positive Phase III lidERA trial for giredestrant represents the first demonstration of a significant IDFS benefit with a novel endocrine therapy in the adjuvant setting since the introduction of aromatase inhibitors. With a 30% reduction in recurrence risk and a favorable tolerability profile, giredestrant is poised to become a new standard backbone therapy for higher-risk ER+/HER2− early breast cancer, pending regulatory approval [4,5,6,8,10].

In HER2-positive disease, the dual impact of T-DXd in both adjuvant (DESTINY-Breast05) and first-line metastatic (DESTINY-Breast09) settings creates a therapeutic continuum that redefines expectations. The 53% reduction in recurrence risk with adjuvant T-DXd establishes a new efficacy benchmark, while the 13.8-month PFS improvement in first-line metastatic disease (40.7 vs. 26.9 months) similarly resets expectations for advanced disease management. These advances necessitate careful consideration of treatment sequencing, particularly regarding the optimal integration of neoadjuvant T-DXd (DESTINY-Breast11) versus postoperative administration. The emergence of next-generation ADCs with distinct toxicity profiles (SHR-A1811 in HORIZON-Breast01) further enriches the therapeutic landscape while introducing new choices regarding toxicity management preferences (Figure 3) [14,15,18,33].

For triple-negative breast cancer, the validation of TROP2-directed ADCs as first-line therapy represents a paradigm shift from chemotherapy-dominated approaches. The concurrent availability of sacituzumab govitecan (ASCENT-03) and datopotamab deruxtecan (BEGONIA) with distinct toxicity profiles (neutropenia/diarrhea vs. stomatitis/ocular toxicity) allows treatment selection based on patient-specific factors and toxicity management capabilities. The confirmation of long-term immunotherapy benefit in early disease (GeparNuevo 7-year follow-up) solidifies the role of immune checkpoint inhibitors, while biomarker refinement (TILs in A-BRAVE) promises more precise patient selection (Figure 4) [20,23,42,44,47].

In HR+/HER2− metastatic disease, the evolution from sequential single-agent therapy to biomarker-guided combination strategies represents perhaps the most sophisticated advancement. The proactive, liquid biopsy-guided intervention paradigm validated in SERENA-6 (PFS HR = 0.44 with ESR1 mutation-directed switching) fundamentally transforms the management approach from reactive to pre-emptive. Concurrently, the validation of strategic sequencing (SONIA supporting delayed CDK4/6 inhibitor use) and novel agent classes (PROTAC degraders in VERITAC-2) creates a multifaceted, personalized approach to overcoming endocrine resistance (Figure 7) [52,54].

The SABCS 2025 data provided critical updates that refine several therapeutic paradigms. In metastatic HR+/HER2− disease, the updated EMBER-3 results reinforce the efficacy of oral SERDs, positioning imlunestrant as a potent option for *ESR1m* patients and a backbone for combination therapy (Figure 7). The mature, pooled analysis on carboplatin in early TNBC resolved lingering questions, providing definitive evidence that pCR benefits translate into survival gains in the neoadjuvant setting and establishing its value in the adjuvant setting for surgically managed patients. In HER2+ metastatic disease, SABCS 2025 clarified the sequencing landscape: HER2CLIMB-05 defined an optimal maintenance strategy post-THP induction, while DESTINY-Breast09 patient-reported outcomes supported the feasibility of shifting to frontline T-DXd-based therapy (Figure 5). For HER2+/HR+ early disease, the ALTTO analysis offers long-awaited guidance, strongly favoring AI + OFS over tamoxifen (Figure 3) [19,33,35,56].

### 4.2. Overarching Themes and Implications

#### 4.2.1. Biomarker-Driven Personalization Across the Continuum

A consistent theme across subtypes is the refinement of biomarker-driven approaches, evolving from static assessment to dynamic monitoring. The progression from single gene testing (ESR1, PIK3CA) to complex signatures (PAM50 ROR in RIBOLARIS), and from tissue-based to liquid biopsy approaches (ctDNA monitoring in SERENA-6), reflects an increasing sophistication in personalization (Figure 1 and Figure 7). Tumor-infiltrating lymphocytes emerging as predictive biomarkers in TNBC (A-BRAVE) further exemplifies this trend toward microenvironment-based assessment (Figure 6). The integration of these biomarkers into clinical practice requires not only laboratory infrastructure but also interpretive expertise and clear clinical algorithms for actionability [6,23,51].

#### 4.2.2. Antibody-Drug Conjugates as Transformative Platform Therapeutics

ADCs have evolved from niche agents to backbone therapies across multiple settings, representing perhaps the most impactful therapeutic platform since the introduction of targeted therapies. Their success raises important questions about optimal sequencing (particularly as they move into earlier disease settings), combination strategies (with immunotherapy, targeted therapies, or other ADCs), and management of unique toxicities (ILD with T-DXd, ocular toxicity with datopotamab deruxtecan, neutropenia with sacituzumab govitecan). The differential toxicity profiles of various ADCs will increasingly influence clinical choice based on patient comorbidities and institutional management capabilities.

The SABCS 2025 focus on structured management algorithms for ADC-associated toxicities, such as ocular events and stomatitis, is a direct response to this clinical need. This underscores that the safe and effective integration of these powerful agents into practice requires not only efficacy data but also the development and dissemination of expert-led management protocols to mitigate unique adverse events and preserve treatment duration.

#### 4.2.3. Strategic Treatment Sequencing and Intelligent De-Escalation

The maturation of evidence supporting strategic de-escalation represents a counterbalance to therapeutic intensification trends. SONIA’s validation of delayed CDK4/6 inhibitor use, RIBOLARIS’s chemotherapy omission based on molecular response, and the reassurance regarding treatment interruption for fertility (POSITIVE) collectively support a more nuanced approach to treatment intensity that considers not only efficacy but also quality of life, long-term toxicity, and patient priorities. This balance of escalation for high-risk disease and de-escalation for favorable scenarios represents a maturation in personalized cancer care (Figure 1 and Figure 7).

#### 4.2.4. Proactive Rather than Reactive Management Paradigms

The SERENA-6 trial paradigm, using liquid biopsy to detect resistance mutations before radiographic progression and proactively switching therapy, represents a fundamental shift in cancer management. This approach, potentially applicable to other resistance mechanisms (PIK3CA mutations, HER2 amplifications), moves precision medicine from static assessment at diagnosis to dynamic monitoring throughout treatment. Implementation challenges include establishing reliable ctDNA testing protocols, determining optimal monitoring intervals, and developing clear clinical pathways for action upon mutation detection (Figure 7) [51].

#### 4.2.5. Global Equity and Access Considerations

The 2025 evidence base presents both challenges and solutions regarding global equity. The high costs of novel therapies (ADCs, CDK4/6 inhibitors) and infrastructure requirements for biomarker testing create implementation challenges, particularly in resource-limited settings. However, trials like PLANET (ultra-low-dose pembrolizumab) and SYSUCC-001 (metronomic capecitabine) demonstrate that cost-effective strategies can maintain meaningful efficacy. The emergence of biosimilar and biobetter versions of established therapies may further improve accessibility in coming years (Figure 3) [24,32].

#### 4.2.6. Patient-Reported Outcomes as Essential Endpoints

The consistent inclusion and reporting of patient-reported outcomes across trials (PATINA, SERENA-6, VERITAC-2) reflects growing recognition that efficacy alone is insufficient; treatments must also preserve or improve quality of life. This is particularly relevant for chronic therapies in metastatic settings and adjuvant treatments with long-term toxicities. The development of standardized PRO assessment methodologies and their integration into regulatory approvals and treatment guidelines will be essential for maintaining this focus [28,52,54].

This was exemplified at SABCS 2025 by the presentation of PRO data from DESTINY-Breast09, which demonstrated comparable tolerability burden between novel ADC-based regimens and traditional chemotherapy from the patient’s perspective. Furthermore, the detailed reporting on quality-of-life impacts in trials like SERENA-6 and the focus on managing treatment-related symptoms (e.g., vasomotor symptoms in OASIS-4) highlight the field’s commitment to a more holistic view of treatment benefit.

### 4.3. Clinical Implementation Challenges

#### 4.3.1. Treatment Sequencing Complexities

The proliferation of effective agents creates increasingly complex sequencing decisions. In HER2-positive disease, the optimal integration of T-DXd across neoadjuvant, adjuvant, and metastatic settings requires careful consideration (Figure 5) [33,39]. In HR+ metastatic disease, the availability of multiple novel endocrine agents (oral SERDs, PROTAC degraders) alongside established CDK4/6 inhibitors and PI3K inhibitors creates decision trees requiring sophisticated biomarker guidance (Figure 7). Development of clinical decision support tools and multidisciplinary tumor boards will be essential for navigating these complexities [52,53,58].

#### 4.3.2. Toxicity Management Specialization

The unique toxicity profiles of novel agents require specialized management expertise. Interstitial lung disease monitoring and management for T-DXd, ocular toxicity prevention for datopotamab deruxtecan, and diarrhea management for sacituzumab govitecan necessitate protocol development and staff education. The creation of toxicity management teams and referral pathways will become increasingly important as these agents move into broader use [33,44].

#### 4.3.3. Biomarker Testing Infrastructure

The implementation of dynamic biomarker monitoring (ctDNA for ESR1 mutations) requires not only laboratory capabilities but also integration into clinical workflows, insurance coverage, and clear clinical action algorithms. Similarly, the move toward complex biomarker signatures (PAM50 ROR, TIL assessment) necessitates standardization of testing methodologies and interpretation criteria across institutions [6,23,51].

#### 4.3.4. Financial Toxicity and Access Barriers

The high costs of novel therapies create significant financial toxicity for patients and healthcare systems. Even in settings with insurance coverage, co-payments and non-medical costs (travel, time off work) can be prohibitive. Development of alternative dosing strategies (as in PLANET), biosimilar competition, and value-based pricing models will be essential for sustainable access [24].

### 4.4. Limitations of the Evidence Base

#### 4.4.1. Interim Nature of Conference Data

Many practice-changing presentations at ASCO and ESMO 2025 were interim analyses, with final data and subsequent publications needed for complete assessment. Overall survival data were immature for several pivotal trials (DESTINY-Breast09, ASCENT-03), necessitating cautious interpretation and potential future refinement of treatment recommendations [33,42].

#### 4.4.2. Generalizability Concerns

Included trials predominantly enrolled patients from high-income countries with standardized healthcare infrastructure. Generalizability to resource-limited settings, diverse ethnic populations, and patients with significant comorbidities is uncertain. Underrepresentation of certain populations (older adults, those with multiple comorbidities) limits understanding of real-world effectiveness and toxicity [24,49].

#### 4.4.3. Publication and Presentation Bias

Positive trials are more likely to be presented at major conferences and published in high-impact journals. Negative or equivocal trials may be under-represented in this synthesis. The curated selection approach, while focusing on practice-changing advances, may overlook important negative results that inform therapeutic boundaries.

#### 4.4.4. Heterogeneity in Endpoints and Assessments

Variability in endpoint definitions, assessment schedules, and response criteria across trials complicates cross-trial comparisons. Differences in blinding procedures, independent review processes, and statistical analysis plans further challenge direct comparison of treatment effects.

#### 4.4.5. Short Follow-Up for Novel Agents

Long-term safety data for recently introduced agents, particularly those with unique mechanisms (PROTAC degraders, novel ADCs), are limited. Chronic or delayed toxicities may emerge with longer follow-up, potentially altering risk-benefit assessments, especially in curative settings [53].

### 4.5. Future Research Directions

#### 4.5.1. Biomarker Refinement and Validation

Priorities include: prospective validation of ctDNA monitoring strategies across subtypes; development of predictive biomarkers for ADC response beyond target expression; integration of multi-omics approaches for comprehensive biomarker discovery; and standardization of biomarker assessment methodologies. Particular attention should focus on biomarkers for de-escalation strategies and for identifying patients unlikely to benefit from specific therapies [51].

#### 4.5.2. Treatment Sequencing and Combination Optimization

Key questions include: optimal sequencing of multiple active agents in HER2-positive disease; rational combination strategies for ADCs with immunotherapy, targeted therapies, or other ADCs; duration of therapy in adjuvant and maintenance settings; and re-challenge strategies after treatment breaks (Figure 5 and Figure 6). Adaptive trial designs that allow within-trial treatment modification based on response will be particularly valuable.

#### 4.5.3. Overcoming Resistance Mechanisms

Research should focus on: understanding and targeting acquired resistance to CDK4/6 inhibitors, ADCs, and novel endocrine agents; developing therapies for biomarker-defined resistant subsets (e.g., RB1-loss, CCNE1-amplified); exploring adaptive therapy approaches to delay resistance; and investigating rechallenge strategies after resistance development.

#### 4.5.4. Global Health and Implementation Science

Critical areas include: development of cost-effective treatment strategies without compromising efficacy; implementation of biomarker testing in low-resource settings; study of novel agents in diverse populations; and integration of palliative care and symptom management. Implementation science research should focus on strategies to reduce disparities in access and outcomes.

#### 4.5.5. Patient-Reported Outcomes and Quality of Life

Future trials should: standardize PRO assessment methodologies; include PROs as primary or co-primary endpoints where appropriate; develop interventions for treatment-related symptoms; and study long-term quality of life in survivorship. Particular attention should focus on cognitive effects, sexual health, and financial toxicity [43,52,54].

#### 4.5.6. Novel Therapeutic Platforms

Emerging platforms requiring investigation include: bispecific and trispecific antibodies; novel ADC payloads and linkers; immune cell engagers; cancer vaccines; and adoptive cell therapies. Combination approaches integrating multiple novel platforms may offer synergistic benefits. The successful outcome of the lidERA trial underscores the potential of next-generation oral SERDs to replace older endocrine therapies. Future research should focus on optimal patient selection (e.g., by genomic risk), combination strategies with CDK4/6 inhibitors (as evaluated in the lidERA substudy), and long-term safety and quality-of-life data [10].

### 4.6. Translation to Guidelines: Categorization of Biomarker-Driven Strategies from Evidence to Practice

A critical distinction emerging from the 2025 evidence base is the maturation of biomarker strategies across three distinct translational stages: those already embedded in clinical guidelines, those with compelling new evidence warranting imminent integration, and those demonstrating promise but remaining investigational. This systematic review identifies clear demarcations that are essential for clinicians interpreting emerging data and implementing precision oncology in routine practice.

#### 4.6.1. Guideline-Integrated Biomarkers (Current Standard of Care)

Several biomarkers have achieved consensus-level recommendation in international guidelines and represent non-negotiable components of contemporary breast cancer management. Estrogen receptor and progesterone receptor status, determined by immunohistochemistry, remain foundational for all stages of breast cancer to guide endocrine therapy allocation. Human epidermal growth factor receptor 2 testing dictates eligibility for anti-HER2 therapies including trastuzumab, pertuzumab, T-DM1, and trastuzumab deruxtecan across adjuvant and metastatic settings. Programmed death ligand 1 (PD-L1) combined positive score (CPS) assessment is guideline-mandated for metastatic TNBC to select patients for first-line pembrolizumab plus chemotherapy [64]. Germline BRCA1/2 mutation testing is established standard of care for both early-stage (adjuvant olaparib for high-risk disease) and metastatic (PARP inhibitor therapy) triple-negative and HER2-negative breast cancers [65,66]. Somatic PIK3CA mutation testing is guideline-recommended for hormone receptor-positive, HER2-negative metastatic breast cancer following progression on endocrine-based therapy to identify candidates for alpelisib plus fulvestrant. *ESR1* mutation testing in circulating tumor DNA is now integrated into guidelines for hormone receptor-positive metastatic breast cancer progressing on endocrine therapy, guiding use of elacestrant in later lines [67]. These biomarkers require no additional validation for implementation and should be universally accessible.

#### 4.6.2. Biomarkers with Practice-Changing 2025 Evidence (Imminent Guideline Integration)

The 2025 trial landscape provides definitive evidence supporting expansion of biomarker-driven strategies into new contexts, with pending regulatory approvals and guideline updates anticipated within 12–24 months. Most transformative is the validation of prospective, serial circulating tumor DNA monitoring for emergent *ESR1* mutations in patients receiving first-line aromatase inhibitor plus CDK4/6 inhibitor for hormone receptor-positive metastatic breast cancer. The SERENA-6 trial establishes that pre-emptive therapy switching to camizestrant upon mutation detection, before radiographic progression, significantly improves PFS (HR = 0.44). This represents the first validated paradigm for proactive, liquid biopsy-guided intervention and is poised for immediate guideline integration contingent upon regulatory approval [51,52]. TROP2 expression assessment, while not yet standardized or mandated, now warrants consideration given the first-line approvals of sacituzumab govitecan (ASCENT-03) and datopotamab deruxtecan (BEGONIA) in metastatic TNBC. Unlike HER2, the binary presence of TROP2 is sufficient for ADC activity, and routine immunohistochemical testing is not currently required for clinical decision-making; however, the extraordinary efficacy observed across unselected populations suggests that treatment allocation currently need not await TROP2 testing [42,43,44].

#### 4.6.3. Promising Biomarkers Under Investigation (Not for Routine Clinical Use)

Several biomarker strategies with compelling biological rationale and supportive retrospective or exploratory analyses require prospective validation before routine adoption. Stromal TILs demonstrate strong prognostic and potentially predictive value in early TNBC, with the A-BRAVE translational analysis suggesting that patients with high baseline sTILs (≥30%) derive substantial benefit from adjuvant avelumab (distant recurrence HR = 0.31). Despite this signal, sTILs are not yet a validated predictive biomarker for immunotherapy selection in routine practice due to absence of prospective trials using sTILs as a randomization or eligibility criterion [23]. Standardized scoring methodologies are well-established, but prospective interventional trials are required for guideline endorsement. ESR1 mutation monitoring in the adjuvant setting, while biologically plausible and technically feasible, lacks interventional trial data demonstrating that early detection and pre-emptive therapy escalation improves cure rates. Several ongoing studies are addressing this question, but current evidence does not support routine ctDNA surveillance in early-stage breast cancer. PAM50-based ROR scores and other genomic signatures, while validated for prognostic stratification and chemotherapy de-escalation in hormone receptor-positive early breast cancer, require further validation for guiding novel agent selection in the neoadjuvant or adjuvant settings as demonstrated in the RIBOLARIS trial [6]. Additional emerging biomarkers including PALB2 mutations, homologous recombination deficiency signatures beyond BRCA1/2, and novel resistance mutations (RB1 loss, CCNE1 amplification, FAT1 alterations) remain firmly investigational and should not influence treatment decisions outside of clinical trial enrollment [29,59,60].

This categorical distinction is essential for clinicians navigating the rapidly expanding biomarker landscape. While 2025 has unquestionably accelerated the translation of precision oncology from concept to clinical reality, the discipline of distinguishing evidence levels remains paramount for responsible, cost-effective, and guideline-concordant cancer care.

## 5. Conclusions

The year 2025 represents a watershed moment in breast oncology, with evidence from major conferences and publications collectively redefining standards of care across all disease stages and molecular subtypes. This systematic review synthesizes these advances into a coherent narrative, highlighting both transformative successes and persistent challenges.

Key 2025 advances include definitive evidence for carboplatin in early TNBC, optimized sequencing and maintenance strategies in HER2+ metastatic disease, practice-guiding insights on endocrine therapy for HER2+/HR+ early cancer, and mature updates supporting novel oral SERDs like imlunestrant.

The maturation of adjuvant CDK4/6 inhibitor data, with proven OS benefit, cements these agents as foundational in high-risk HR+ early breast cancer. The ascendance of antibody-drug conjugates, particularly trastuzumab deruxtecan in HER2-positive disease and TROP2-directed agents in triple-negative breast cancer, has created new efficacy benchmarks while introducing unique management considerations. Perhaps most innovatively, the validation of proactive, liquid biopsy-guided therapy switching in metastatic HR+ disease represents a paradigm shift toward pre-emptive precision medicine.

Unifying themes emerge across subtypes: the critical importance of biomarker-driven personalization; the balance between treatment escalation for high-risk disease and intelligent de-escalation when possible; the growing emphasis on patient-reported outcomes and quality of life; and the urgent need to address global equity in cancer care.

Implementation of these advances will require multidisciplinary collaboration, education on new toxicity management, development of biomarker testing infrastructure, and attention to financial toxicity. Guideline bodies must rapidly integrate this evidence, while healthcare systems must address access disparities. As the breast oncology community looks beyond 2025, research priorities include: refining predictive biomarkers, understanding and overcoming resistance mechanisms, optimizing treatment sequencing and combinations, and ensuring equitable global access.

The remarkable progress chronicled in this review provides both powerful new tools and a roadmap for their optimal application in the ongoing pursuit of personalized, effective, and compassionate breast cancer care. The challenge now lies not in developing more effective therapies, but in wisely integrating the existing arsenal to maximize benefit while minimizing harm, always keeping the patient experience at the center of therapeutic decision-making. The year 2025 also delivered practice-changing adjuvant data for the oral SERD giredestrant (lidERA trial), which significantly improved invasive disease-free survival in ER+/HER2− early breast cancer. This milestone, along with the maturation of adjuvant CDK4/6 inhibitor data and the ascendance of ADCs, collectively redefines curative-intent treatment paradigms.

## Figures and Tables

**Table 1:** PICO framework for study inclusion.

Element	Inclusion Criteria
Population	Adult patients (≥18 years) with histologically confirmed breast cancer of any stage (early or metastatic).
Intervention	Systemic therapies including: chemotherapy, endocrine therapy, targeted agents (e.g., HER2, TROP2, CDK4/6, PI3K, PARP, mTOR inhibitors), immunotherapy, ADCs, and novel mechanism agents (e.g., PROTAC degraders, oral SERDs, bispecific antibodies).
Comparison	Standard of care treatment, placebo, best supportive care, or active comparator (e.g., different drug or combination). Single-arm studies were considered only if they represented the only available evidence for a novel agent in a specific context.
Outcome	Primary efficacy endpoints: OS, PFS, IDFS, pCR, ORR. Secondary endpoints included safety, duration of response, and PROs.

Table abbreviations: ADC: Antibody-Drug Conjugate, CDK4/6, Cyclin-Dependent Kinase 4/6HER2: HER 2; IDFS: Invasive Disease-Free Survival; mTOR: Mammalian Target of Rapamycin; ORR: Objective Response Rate; OS: Overall Survival; PARP: Poly(ADP-Ribose) Polymerase; pCR: Pathological Complete Response; PFS: Progression-Free Survival; PI3K: Phosphatidylinositol 3-Kinase; PRO: Patient-Reported Outcome; PROTAC: Proteolysis-Targeting Chimera; SERD: Selective Estrogen Receptor Degrader; TROP2: Trophoblast Cell-Surface Antigen 2.

**Table 2:** Summary of Pivotal Breast Cancer Clinical Trials in 2025. This comprehensive table provides an overview of the design, population, interventions, efficacy outcomes, and safety results for the 50 Phase II/III randomized controlled trials included in this systematic review.

Trial Name/Reference	Phase	Population (Subtype, Stage)	Experimental Arm	Control Arm	Primary Endpoint (s)	Key Efficacy Results	Key Safety/Tolerability Results
HORMONE RECEPTOR-POSITIVE (HR+), HER2-NEGATIVE (18)							
monarchE (ESMO 2025) [4]	III	HR+/HER2− Early BC, node-positive, high-risk	Abemaciclib (2 y) + ET	ET alone	iDFS, OS	6.3-yr OS: HR 0.842 (95% CI: 0.722–0.981; *p* = 0.0273). 5-yr OS: 86.8% vs. 85%. iDFS: HR 0.734.	Diarrhea, neutropenia. No new delayed toxicity signals.
NATALEE (ESMO 2025) [5]	III	HR+/HER2− Early BC (Stage II/III)	Ribociclib (400 mg, 3 y) + NSAI (5 y)	NSAI alone (5 y)	iDFS	5-yr iDFS: 85.5% vs. 81% (delta 4.5, HR 0.716). OS trend favorable.	Consistent with known ribociclib profile.
RIBOLARIS (ESMO 2025) [6]	II	HR+/HER2− Early BC, clinically high-risk	Neoadj. Ribociclib + Letrozole → ROR-guided adj. therapy	-	DMFS in ROR-low	52.6% achieved low ROR, omitting chemo. Neoadj. progression: 2.19%.	Neutropenia, liver enzyme elevations.
POSITIVE (ESMO 2025) [7]	Cohort	HR+/HER2− Early BC, ≤42 yrs, desire for pregnancy	Temporary ET interruption (≤2 y) for pregnancy attempt	Matched cohort (SOFT/TEXT)	BCFI	5-yr BCFI: 87.7% vs. 86.8%, HR vs. SOFT/TEXT: 0.88 (0.66–1.18). Pregnancy rate: 76%.	Safe interruption; 82% resumed ET.
EMPRESS (ESMO 2025) [8]	II	HR+/HER2− Early BC, premenopausal, Ki67 ≥ 10%	Giredestrant (15 d pre-op)	Tamoxifen (15 d pre-op)	Ki67 change	Relative Ki67 reduction: −73% vs. −51% (*p* < 0.001). Cell cycle arrest: 17.5% vs. 4.5% (*p* = 0.074).	Fatigue, hot flush. Low TRAEs (31.0% vs. 38.6%).
TACTIVE-N (ESMO 2025) [9]	II	HR+/HER2− Early BC, postmenopausal, treatment-naïve	Vepdegestrant (neoadjuvant)	Anastrozole (neoadjuvant)	Ki67 change at D15	Ki67 reduction: −71.4% vs. −72.9%. mPEPI 0: 21% vs. 20%. BCS rate: 70% vs. 54%.	Hot flashes, asthenia. Low discontinuation (3% vs. 8%).
lidERA (SABCS 2025) [10]	III	ER+/HER2− Early BC, higher-risk (Stage I–III)	Adjuvant Giredestrant (oral, 30 mg QD)	Adjuvant Standard ET (Tamoxifen or AI)	IDFS (excluding second primary non-breast cancer)	IDFS HR = 0.70 (95% CI: 0.57–0.87; *p* = 0.0014). 3-year IDFS: 92.4% vs. 89.6%. DRFI HR = 0.69 (95% CI: 0.55–0.87). Interim OS HR = 0.79 (95% CI: 0.56–1.12).	Favorable safety profile. Lower discontinuation rate (5.3% vs. 8.2%). Lower rate of discontinuations due to musculoskeletal (1.8% vs. 4.4%) and vasomotor (<0.1% vs. 0.9%) disorders. Comparable rates of Grade 3–4 AEs (19.8% vs. 17.9%).
SOFT/TEXT 15-yr Update (ASCO 2025) [11,12]	III	Premenopausal HR+ eBC	SOFT: T + OFS or E + OFS vs. T; TEXT: E + OFS vs. T + OFS	SOFT: T alone; TEXT: T + OFS	BCFI, DRFI, OS	SOFT: BCFI E + OFS vs. T: 78.6% vs. 72.1% (HR 0.70). TEXT/Combined: DRFI benefit for E + OFS vs. T + OFS (87.6% vs. 83.7% HR 0.75). OS benefit in high-risk (79.4% vs. 75.6%).	Long-term safety as expected.
OASIS-4 (ASCO 2025) [13]	III	HR+/HER2− eBC with VMS on ET	Elinzanetant 120 mg daily	Placebo	Mean change in daily frequency of moderate-to-severe VMS (wks 4–12)	Mean difference vs. placebo: −3.5 (*p* < 0.0001). Significant decrease in severity.	Fatigue (9.5%), somnolence (10%), diarrhea (5.1%). Well tolerated.
SONIA (ESMO 2025) [49]	III and Economic	HR+/HER2− ABC	Strategy A: CDK4/6i + AI 1 L → Fulvestrant 2 L	Strategy B: AI 1 L → CDK4/6i + Fulvestrant 2 L	PFS2, QALYs	No sig. diff in PFS2. OS: 47.9 vs. 48.1 mo (HR 0.91, *p* = 0.24). QALYs: 2.694 vs. 2.644.	74% more Gr3–4 AEs with 1 L CDK4/6i.
CULMINATE-2 (ESMO 2025) [50]	III	HR+/HER2− ABC, pretreated	Culmerciclib (CDK2/4/6i) + Fulvestrant	Placebo + Fulvestrant	PFS	Median PFS: NR vs. 20.2 mo (HR 0.56, *p* = 0.0004). ORR: 59.3% vs. 42.3% (*p* = 0.0009).	Gr ≥ 3 neutropenia 20.3%, leukopenia 10.7%. Low discontinuation (3.5%).
SERENA-6 (ASCO 2025) [51,52]	III	HR+/HER2− ABC with emergent ESR1m on 1 L AI + CDK4/6i	Switch to Camizestrant + CDK4/6i	Continue AI + CDK4/6i	PFS	Median PFS: 16.0 vs. 9.2 mo (HR 0.44, *p* < 0.0001).	Delayed TTD in GHS/QoL (HR 0.54), pain (HR 0.57). Modest neutropenia increase.
VERITAC-2 (ASCO 2025) [53,54]	III	ER+/HER2− ABC with ESR1m; post-ET + CDK4/6i	Vepdegestrant	Fulvestrant	PFS	PFS benefit met	Favorable safety; low GI AEs, low discontinuations. Superior PROs.
evERA BC (ESMO 2025) [55]	III	ER+/HER2− aBC (1–3 L); post-CDK4/6i; 55% ESR1m	Giredestrant + Everolimus	SOC ET + Everolimus	INV-PFS (ESR1m & ITT)	ESR1m: PFS 9.99 vs. 5.45 mo (HR 0.38, *p* < 0.0001). ITT: 8.77 vs. 5.49 mo (HR 0.56, *p* < 0.0001).	Stomatitis, diarrhea, anemia. Manageable profile.
EMBER-3 (SABCS 2025) [56]	III	ER+/HER2− ABC post-ET	1. Imlunestrant mono 2. Imlunestrant + Abemaciclib	SOC ET (Fulv/Exe)	PFS, OS (key updates)	ESR1m: OS Δ +11.4 mo (HR = 0.60, *p* = 0.0043); PFS HR = 0.62. All: Imlunestrant + Abema PFS HR = 0.58 (10.9 vs. 5.5 mo); OS trend HR = 0.82.	Favorable profile. Combo: Low D/C rate (6%). No new oral SERD-specific signals.
INAVO120 (ASCO 2025) [57]	III	HR+/HER2− ABC with PIK3CA mutation, post-CDK4/6i	Inavolisib + Palbociclib + Fulvestrant	Placebo + Palbociclib + Fulvestrant	PFS	Median PFS: 15.0 vs. 7.3 mo (HR 0.43). ORR: 58% vs. 25%. Final OS: 34.0 vs. 27.0 mo (HR 0.67, *p* = 0.019).	Hyperglycemia, stomatitis, diarrhea, rash.
VIKTORIA-1 (ESMO2025) [58]	III	HR+/HER2− ABC, PIK3CA-WT, post-CDK4/6i	Gedatolisib + Fulvestrant ± Palbociclib	Fulvestrant	PFS	Triplet: PFS 9.3 vs. 2.0 mo (HR 0.24). Doublet: 7.4 vs. 2.0 mo (HR 0.33). ORR: 31.5%/28.3% vs. 1.0%.	Stomatitis, neutropenia, nausea. Low Gr3 hyperglycemia (2.3%).
DOLAF (SABCS 2025) [63]	II	ER+/HER2− mBC with genomic alterations (e.g., gBRCAm), 2nd/3rd line	Durvalumab + Olaparib + Fulvestrant	-	24-week PFS rate	24-wk PFS rate: 66.7% (ITT), 76.3% in gBRCAm. Median PFS: 9.3 mo (ITT).	Nausea (59%), asthenia (43%). Acceptable.
HER2-POSITIVE (13)							
DESTINY-Breast05 (ESMO 2025) [14]	III	HER2+ Early BC with residual disease post-neoadjuvant	Adjuvant T-DXd (14 cycles)	Adjuvant T-DM1 (14 cycles)	IDFS	3-yr IDFS: 92.4% vs. 83.7% (HR 0.47, *p* < 0.0001). Benefit across subgroups.	ILD: 9.6% (any grade) vs. 1.6%. Includes 0.9% Gr ≥ 3, 0.2% fatal.
DESTINY-Breast11 (ESMO 2025) [15]	III	High-risk HER2+ Early BC (cT3-4/N+)	T-DXd → THP (neoadjuvant)	ddAC-THP (neoadjuvant)	pCR	pCR: 67.3% vs. 56.3% (Δ11.2%, *p* = 0.003). Benefit in HR− (Δ16.1%).	Fewer Gr ≥ 3 AEs (37.5% vs. 55.8%), serious AEs, LV dysfunction (1.3% vs. 6.1%). ILD ~4–5%.
neoCARHP (ASCO 2025) [16]	III	HER2+ Early BC (Stage II–III)	THP (6 cycles)	TCbHP (6 cycles)	pCR	pCR: 64.1% vs. 65.9% (non-inferior).	Lower Gr3/4 neutropenia (6.8% vs. 16.4%), anemia (2.1% vs. 6.6%), nausea/vomiting.
TQB2102 (PUBMED 2025) [17]	II (Rand)	HER2+ Stage II–III BC (Neoadjuvant)	TQB2102 (bispecific ADC)	Historical control (40% tpCR threshold)	tpCR rate	tpCR rates: 57.7% to 76.9% across cohorts. All exceeded 40% threshold.	Gr ≥ 3 TRAEs: 23.1–30.8%. No treatment-related deaths.
SHR-A1811 ± Pyrotinib (SABCS 2025) [18]	II	Stage II–III HER2+ BC (Neoadjuvant)	SHR-A1811 (ADC) mono or + Pyrotinib vs. PChHP	-	pCR	pCR: 63.2% (mono), 62.5% (combo), 64.4% (PChHP). Robust ADC activity.	Gr ≥ 3 AEs: 44.8% (mono), 71.6% (combo). One G2 ILD in ADC arm.
ALTTO Analysis (SABCS 2025) [19]	Explor. (III)	HER2+/HR+ Early BC post-chemo & 1 y anti-HER2	Adjuvant AI (+OFS if premenopausal)	Adjuvant Tamoxifen (± OFS)	DFS (exploratory)	AI ± OFS vs. Tamoxifen: DFS HR = 0.65. Premenopausal: AI + OFS vs. Tamoxifen HR = 0.44 (10-yr DFS 90.0% vs. 77.6%).	As expected for AI vs. Tamoxifen.
DESTINY-Breast09 (ASCO 2025) [33,34]	III	HER2+ Metastatic BC, first-line	T-DXd ± Pertuzumab	Taxane + Trastuzumab + Pertuzumab	PFS, OS PROs (PGI-TT)	PFS: 40.7 vs. 26.9 mo (HR 0.58, *p* < 0.00001). ORR: 89.9% vs. 80.3%. OS interim: HR 0.74. PROs: Similar overall tolerability burden between T-DXd + P and THP per patient report (PGI-TT).	ILD: 12% in T-DXd arms (majority low grade). Higher GI toxicity vs. THP. Different toxicity profiles (ILD/nausea vs. chemo side effects) but comparable patient-reported bother.
HER2CLIMB-05 (SABCS 2025) [35]	III	HER2+ MBC, no progression after 4–8 cycles of 1 L THP	Maintenance: Tucatinib + Trastuzumab + Pertuzumab	Maintenance: Placebo + Trastuzumab + Pertuzumab	PFS	Median PFS: 24.9 vs. 16.3 mo (HR 0.58, *p* < 0.0001). Benefit across subgroups (HR− and HR+).	Safety consistent with known tucatinib profile (diarrhea, hepatotoxicity).
HORIZON-Breast01 (ESMO 2025) [36]	III	HER2+ ABC, post-taxane & trastuzumab	SHR-A1811	Pyrotinib + Capecitabine	PFS	Median PFS: 30.6 vs. 8.3 mo (HR 0.22, *p* < 0.0001). ORR: 81.7% vs. 55.9%. OS interim: HR 0.31.	Hematologic toxicities predominant; ILD 2.8% (0.7% Gr ≥ 3).
PATINA (ESMO 2025) [38]	III	HR+/HER2+ MBC, post-induction chemo	Palbociclib + anti-HER2 + ET	anti-HER2 + ET	PFS	Median PFS: 44.3 vs. 29.1 mo (HR 0.74, *p* = 0.0109).	Higher Gr ≥ 3 neutropenia, diarrhea, fatigue. HRQoL preserved.
DESTINY-Breast04 (PUBMED 2025) [39]	III	HER2-low (IHC 1+ or 2+/ISH-) mBC, after 1–2 prior chemos	Trastuzumab Deruxtecan (T-DXd)	Physician’s Choice Chemotherapy	PFS (BICR) in HR+ cohort	Median OS: 22.9 vs. 16.8 mo (HR 0.69). Median PFS: 9.9 vs. 5.1 mo (HR 0.50).	ILD/pneumonitis: 12.1% (Gr ≥ 3: 0.8%). Nausea (73%), fatigue (48%).
SGNTUC-019 (PUBMED 2025) [40]	II Basket	HER2-mutated mBC (HER2-negative by IHC)	Tucatinib + Trastuzumab (± Fulvestrant if HR+)	-	ORR	ORR: 41.9%. Median PFS: 9.5 mo. Active in HER2-mutated, IHC-negative disease.	No new safety signals.
Zanidatamab + Docetaxel PUBMED 2025) [41]	Ib/II	HER2+ ABC (first-line)	Zanidatamab + Docetaxel	-	ORR, Safety	Confirmed ORR: 90.9%. Median PFS: 22.1 mo; Median OS: 36.9 mo.	Gr ≥ 3 TEAEs: 71.1% (neutropenia 34%, diarrhea 13%).
TRIPLE-NEGATIVE BREAST CANCER (TNBC) (17)							
GeparNuevo (Long-term) (ESMO 2025) [20]	II	Early TNBC (Stage II–III)	Durvalumab + NACT (no carbo)	NACT alone	pCR/iDFS	7-yr iDFS: 73.7% vs. 60.7% (HR 0.56). 7-yr OS: 91.6% vs. 74.7% (HR 0.33). Benefit irrespective of pCR.	Acceptable tolerance, no new safety signals.
PLANET Trial (ESMO 2025) [24]	II	Stage II–III TNBC	Neoadj. CT + ultra-low-dose Pembro (50 mg q6w, 3 doses)	Neoadj. CT alone	pCR	pCR: 53.8% vs. 40.5% (*p* = 0.047). Absolute Δ +13.3%. RCB 0/1: 71.6% vs. 61.0%.	Lower Gr ≥ 3 AEs (50.0% vs. 59.5%). One treatment-related death (toxic epidermal necrolysis) in Pembro arm.
NRG-BR003 (ASCO 2025) [25]	III	Early TNBC (node+ or high-risk node-)	DD AC → WP + Carboplatin	DD AC → WP	IDFS	5-yr IDFS: 82.7% vs. 77.8% (HR 0.78, *p* = 0.12). OS: 84.4% vs. 87.7% (HR 0.81, *p* = 0.16).	Higher Gr3/4 AEs, hematologic toxicity, anemia, cytopenia with carbo.
Carboplatin Meta-Analysis (SABCS 2025) [26]	Meta-analysis	Early TNBC (Neoadjuvant)	CT + Carboplatin	CT alone	pCR, EFS	pCR: +16.1% (55.0% vs. 38.9%). EFS: 5-yr +7% (74% vs. 67%, HR = 0.70). Benefit regardless of BRCA status.	Increased hematologic toxicity.
RJBC-1501 (SABCS 2025) [27]	III	Stage I–III TNBC (Adjuvant, post-surgery)	EC-TCb	EC-T	DFS	5-yr DFS: 93.1% vs. 89.8% (HR = 0.66, *p* = 0.03). 5-yr Distant DFS: 92.0% vs. 87.8% (HR = 0.66).	Manageable toxicity profile.
CITRINE (SABCS 2025) [28]	III	Node+/high-risk node-negative TNBC (Adjuvant)	DD EC → WP + Carbo	DD EC → WP	DFS	3-yr DFS: 92.3% vs. 85.8% (HR = 0.64). Benefit strongest in 1st year (HR = 0.31).	Increased but manageable hematologic toxicity.
TBCRC-056 (SABCS 2025) [29]	II	gBRCA1/2 or PALB2m, HER2− Early BC (Neoadjuvant)	Niraparib + Dostarlimab (chemo-free)	-	pCR	TNBC Cohort: pCR rate 50% (23/46). RCB 0/1 rate: 60%. No difference with niraparib lead-in.	Safety consistent with known profiles of each agent.
OlympiaN (SABCS 2025) [30]	II	gBRCA, ER− ≤ 10%, HER2− Early BC	Olaparib ± Durvalumab (chemo-free, risk-adapted)	-	pCR	Trial ongoing; design presented at SABCS 2025.	Aims to validate a chemotherapy-sparing, risk-adapted approach.
NeoSTAR (ASCO 2025) [31]	II	Early TNBC (≥T2 and/or N+)	SG + Pembro (4 cycles) → response-guided therapy	-	pCR after 4 cycles SG + Pembro	pCR after 4 cycles: 32% (60% in mBRCA). pCR after SG + Pembro ± additional CT: 50%.	18-mo EFS 90.6%. Radiological RR 66% (30% CR, 36% PR).
SYSUCC-001 (PUBMED 2025) [32]	III	Early-stage TNBC post-standard adjuvant therapy	Metronomic Capecitabine (1 year)	Observation	DFS	10-yr DFS: 78.1% vs. 66.6% (HR 0.61). FOXC1-high tumors derived significant OS benefit.	Long-term safety consistent with known capecitabine profile.
BEGONIA (Arms 7 and 8) (ESMO 2025) [44]	Ib/II	1 L a/mTNBC (any PD-L1)	Dato-DXd + Durvalumab	-	Safety and ORR	Arm 7 (any PD-L1): ORR 79.0%; mPFS 14.0 mo; mDOR 17.6 mo.	Stomatitis (64–82%), nausea, alopecia, dry eye. ILD low (5–9%, no Gr ≥ 3).
TROPION-Breast01 (PUBMED 2025) [45]	III	HR+/HER2− mBC, post-ET and chemo	Datopotamab Deruxtecan (Dato-DXd)	Investigator’s Choice Chemotherapy	PFS (BICR), OS	Median PFS: 6.9 vs. 4.9 mo (HR 0.63). No OS difference (18.6 vs. 18.3 mo).	Stomatitis, nausea, fatigue, alopecia.
A-BRAVE (ESMO 2025) [23]	III	Early, high-risk TNBC after (neo)adjuvant chemo	Avelumab (1 year)	Observation	DFS	No significant DFS improvement (HR 0.81). Descriptive OS benefit (HR 0.66).	Not detailed.
ASCENT-03 (ESMO 2025) [42,43]	III	Untreated advanced TNBC, not candidates for PD-1/L1 inhibitors	Sacituzumab Govitecan (SG)	Chemotherapy (paclitaxel, nab-paclitaxel, or gem/carbo)	PFS (BICR)	Median PFS: 9.7 vs. 6.9 mo (HR 0.62). ORR: 48% vs. 46%.	Gr ≥ 3 AEs: 66% vs. 62% (neutropenia 43% vs. 41%).
ASCENT-4/KEYNOTE-D19 (ASCO 2025) [46]	III	Previously untreated PD-L1-positive advanced triple-negative breast cancer (TNBC)	Sacituzumab Govitecan + Pembrolizumab	Chemotherapy (paclitaxel, nab-paclitaxel, or gemcitabine/carboplatin) + Pembrolizumab	PFS by BICR	Median PFS: 11.2 vs. 7.8 months (HR = 0.65; 95% CI: 0.52–0.81; *p* < 0.001). Objective Response Rate (ORR): 55% vs. 47%.	Grade ≥ 3 adverse events: 68% vs. 65%. Neutropenia: 45% vs. 38%; Diarrhea: 12% vs. 5%. Treatment discontinuation due to adverse events: 8% vs. 6%.
OptiTROP-Breast01 (PUBMED 2025) [47]	III	mTNBC, ≥2 prior lines	Sacituzumab Tirumotecan (TROP2-ADC)	Chemotherapy	PFS (BICR)	Median PFS: 6.7 vs. 2.5 mo (HR 0.32, *p* < 0.00001). OS improved.	Hematologic toxicity frequent.
FABULOUS (PUBMED 2025) [48]	III	HER2− mBC with germline BRCA1/2 mutations	A: Fuzuloparib + Apatinib; B: Fuzuloparib	C: Chemotherapy	PFS (BICR)	Median PFS: A: 11.0, B: 6.7, C: 3.0 mo. A vs. C: HR 0.27.	Gr3–4: neutropenia, anemia, hypertension. 1 treatment-related death (B).
PREVENTION/OTHER (2)							
LIBER (PUBMED 2025) [61]	III	Postmenopausal women with gBRCA1/2 mutations	Letrozole (5 years)	Placebo	5-year incidence of invasive BC	Non-significant trend favoring letrozole (7.8% vs. 13.1%; HR 0.70, *p* = 0.416).	Safety and QoL did not differ statistically.
Tam-01 (PUBMED 2025) [62]	III	Breast intraepithelial neoplasia	Low-dose Tamoxifen (5 mg/d)	Placebo	Reduction in breast events	42% reduction in breast events (HR 0.58).	Limited toxicity.

Table abbreviations: ABC: Advanced Breast Cancer; ADC: Antibody-Drug Conjugate; AE: Adverse Event; AI: Aromatase Inhibitor; BCFI: Breast Cancer-Free Interval; BCS: Breast-Conserving Surgery; BICR: Blinded Independent Central Review; Carbo: Carboplatin; CDK4/6i: CDK4/6 Inhibitor; CT: Chemotherapy; DD: Dose-Dense; DD AC: Dose-Dense Doxorubicin/Cyclophosphamide; DFS: Disease-Free Survival; DOR: Duration of Response; DRFI: Distant Recurrence-Free Interval; EC: Epirubicin/Cyclophosphamide; ET: Endocrine Therapy; EFS: Event-Free Survival; GHS/QoL: Global Health Status/Quality of Life; HR: Hazard Ratio; HRQoL: Health-Related Quality of Life; IDFS: Invasive Disease-Free Survival; ILD: Interstitial Lung Disease; ITT: Intent-To-Treat; LV: Left Ventricular; mBC: Metastatic Breast Cancer; mPEPI: Preoperative Endocrine Prognostic Index; NACT: Neoadjuvant Chemotherapy; NSAI: Non-Steroidal Aromatase Inhibitor; ORR: Objective Response Rate; OS: Overall Survival; PChHP: Paclitaxel/Carboplatin/Trastuzumab/Pertuzumab; pCR: Pathological Complete Response; Pembro: Pembrolizumab; PFS: Progression-Free Survival; PRO: Patient-Reported Outcome; RCB: Residual Cancer Burden; ROR: Risk of Recurrence; SG: Sacituzumab Govitecan; TCb: Taxane/Carboplatin; TCbHP: Docetaxel/Carboplatin/Trastuzumab/Pertuzumab; T-DM1: Trastuzumab Emtansine; T-DXd: Trastuzumab Deruxtecan; TEAE: Treatment-Emergent Adverse Event; THP: Docetaxel/Trastuzumab/Pertuzumab; TNBC: Triple-Negative Breast Cancer; tpCR: total pathological Complete Response; TRAE: Treatment-Related Adverse Event; TTD: Time to Deterioration; VMS: Vasomotor Symptoms; WP: Weekly Paclitaxel; WP: Weekly Paclitaxel.

## Data Availability

All data generated or analyzed during this study are included in this published article. The datasets supporting the conclusions of this article are derived from publicly available sources: conference abstracts and presentations from the ASCO 2025 Annual Meeting, ESMO 2025 Congress, and SABCS 2025, as well as published articles in peer-reviewed journals as referenced. The full search strategy and data extraction forms are available from the author upon reasonable request.

## Supplementary Materials

The following supporting information can be downloaded at: https://www.mdpi.com/article/10.3390/ijms27041971/s1.
