# Supplementary material for: A Systematic Review of Major Advances in Breast Cancer Therapeutics in 2025: Synthesis of Conference and Published Evidence
Source: Int J Mol Sci. 2026 Feb 19;27(4):1971. doi: 10.3390/ijms27041971 (PMC12941040; doi:10.3390/ijms27041971)
Supplement: Supplementary file 1 [file ijms-27-01971-s001.zip › ijms-4144962-supplementary.pdf]

PRISMA 2020 Checklist for Systematic Reviews

Manuscript Title: A Systematic Review of Major Advances in Breast Cancer Therapeutics in 2025: Synthesis of Conference and Published Evidence
Corresponding Author: Prof. Nabil Ismaili
Journal: International Journal of Molecular Sciences
Special Issue: Advances in Molecular Pathology and Treatment of Breast Cancer

SECTION 1: TITLE AND ABSTRACT

tem #	PRISMA 2020 Item Description	Location in Manuscript / Response
**1**	**Title** - Identify the report as a systematic review.	**Title:** "A Systematic Review of Major Advances in Breast Cancer Therapeutics in 2025: Synthesis of Conference and Published Evidence"
**2**	**Abstract** - See the PRISMA 2020 for Abstracts checklist.	**Section:** *Abstract* (Background, Objectives, Methods, Results, Conclusion). The abstract structure follows PRISMA for Abstracts.

SECTION 2: INTRODUCTION

Item #	PRISMA 2020 Item Description	Location in Manuscript / Response
**3**	**Rationale** - Describe the rationale for the review in the context of existing knowledge.	**Section 1.1 and 1.2:** "The Global Burden and Evolving Landscape of Breast Cancer" and "2025: A Watershed Year in Breast Oncology". Describes the need to synthesize dispersed evidence from 2025.
**4**	**Objectives** - Provide an explicit statement of the objective(s) or question(s) the review addresses.	**Section 1.4:** "Rationale and Objectives of This Systematic Review". The four primary objectives are explicitly stated.

SECTION 3: METHODS

Item #	PRISMA 2020 Item Description	Location in Manuscript / Response
**5**	**Eligibility criteria** - Specify the inclusion and exclusion criteria for the review and how studies were grouped for the syntheses.	**Section 2.2:** "Eligibility Criteria". Detailed criteria for study designs, population, interventions, comparators, outcomes, and timeframe. Also summarized in **Table 1 (PICO Framework)**.
**6**	**Information sources** - Specify all databases, registers, websites, organisations, reference lists and other sources searched or consulted to identify studies. Specify the date when each source was last searched.	**Section 2.3.1 and 2.3.2:** Details searches of ASCO 2025, ESMO 2025, SABCS 2025 conference proceedings and PubMed/MEDLINE/Scopus/Embase for 2025 publications.
**7**	**Search strategy** - Present the full search strategies for all databases, registers and websites, including any filters and limits used.	**Referenced as:** "Supplementary File 1". The complete search strategy is provided in a supplementary file.
**8**	**Selection process** - Specify the methods used to decide whether a study met the inclusion criteria of the review, including how many reviewers screened each record and each report retrieved, whether they worked independently, and any automation tools used.	**Section 2.4:** "Study Selection Process". Describes independent screening by two reviewers (Prof. Ismaili & Prof. El Majjaoui), use of Covidence software, and process for resolving disagreements.
**9**	**Data collection process** - Specify the methods used to collect data from reports, including how many reviewers collected data from each report, whether they worked independently, any processes for obtaining or confirming data from study investigators.	**Section 2.4.2:** Describes the full-text review process. **Section 2.5.1:** Describes the standardized data extraction form and process performed by two independent reviewers.
**10**	**Data items** - List and define all outcomes for which data were sought. Specify whether all results that were compatible with each outcome domain in each study were sought,	**Section 2.2.4 and 2.5.1:** Lists primary (OS, PFS, IDFS, pCR, ORR) and secondary outcomes. The standardized extraction form captured all pre-specified outcomes.

Item #	PRISMA 2020 Item Description	Location in Manuscript / Response
	and if not, the methods used to decide which results to collect.	
**11**	**Study risk of bias assessment** - Specify the methods used to assess risk of bias in the included studies, including details of the tool(s) used, how many reviewers assessed each study and whether they worked independently.	**Section 2.5.2:** "Risk of Bias Assessment". Describes use of the revised Cochrane Risk of Bias tool (RoB 2) applied independently by two reviewers to the 34 Phase III trials.
**12**	**Effect measures** - Specify for each outcome the effect measure(s) (e.g. risk ratio, mean difference) used in the synthesis or presentation of results.	**Throughout Results (Section 3):** Effect measures are consistently reported as Hazard Ratios (HR) for time-to-event outcomes and percentages for response rates (e.g., pCR, ORR), with 95% confidence intervals and p-values where available.
**13**	**Synthesis methods** - Describe the processes used to decide which studies were eligible for each synthesis, the methods used to tabulate/display results, and any synthesis methods.	**Section 2.5.3:** "Data Management and Synthesis". Describes the narrative synthesis approach, organized thematically by disease stage and molecular subtype, due to heterogeneity.
**14**	**Reporting bias assessment** - Describe any methods used to assess risk of bias due to missing results in a synthesis (arising from reporting biases).	**Acknowledged in Section 4.4:** "Publication and Presentation Bias" is discussed as a limitation. Formal assessment was not performed due to the narrative synthesis approach and focus on major conferences/journals.
**15**	**Certainty assessment** - Describe any methods used to assess certainty (or confidence) in the body of evidence for an outcome.	**Section 2.6:** "Evidence Grading and Clinical Relevance Assessment". Describes informal grading based on study design, risk of bias, consistency, precision, and directness.

SECTION 4: RESULTS

Item #	PRISMA 2020 Item Description	Location in Manuscript / Response
**16**	**Study selection** - Describe the results of the search and selection process, from the number of records identified in the search to the number of studies included in the review, ideally using a flow diagram.	**Section 3.1:** "Study Selection and Characteristics". **Figure 1:** PRISMA 2020 flow diagram. **Table 2:** PRISMA Flow Summary table with numerical breakdown.
**17**	**Study characteristics** - Cite each included study and present its characteristics.	**Section 3.1 and Table 1:** Provides an overview of the 46 included trials, with detailed characteristics for each pivotal trial presented in the narrative results (Sections 3.3.1 to 3.3.7) and summarized in the comprehensive **Table 1 (Summary of Pivotal Trials)**.
**18**	**Risk of bias in studies** - Present assessments of risk of bias for each included study.	**Section 3.3:** "Risk of Bias Assessment". Reports that among the 34 Phase III RCTs assessed with RoB 2, 22 (65%) had low risk, 8 (24%) had some concerns, and 4 (12%) had high risk of bias.
**19**	**Results of individual studies** - For all outcomes, present for each study: (a) summary statistics for each group and (b) an effect estimate and its precision, ideally using structured tables or plots.	**Sections 3.4.1 to 3.4.7:** For each included trial, key efficacy results (HR, CI, p-values, median values) and safety outcomes are reported. **Table 1** provides a structured summary for all included trials.
**20**	**Results of syntheses** - Present results of all statistical syntheses conducted. If meta-analysis was done, present for each the summary estimate and its precision. If comparing groups, describe the direction of the effect.	**Sections 3.4.1 to 3.4.7 and 4.1:** A narrative synthesis is presented, organized by disease stage and subtype. A meta-analysis was not performed due to heterogeneity. Key findings and effect estimates from individual studies are synthesized thematically.
**21**	**Reporting biases** - Present assessments of risk of bias due to missing results (arising from reporting biases).	**Section 4.4 (Limitations):** "Publication and Presentation Bias" is discussed as a potential limitation, acknowledging that positive trials are more likely to be presented/published.

Item #	PRISMA 2020 Item Description	Location in Manuscript / Response
**22**	**Certainty of evidence** - Present assessments of certainty (or confidence) in the body of evidence for each outcome assessed.	**Integrated into the Discussion (Section 4):** The strength of the evidence is discussed narratively within the synthesis of each major finding, considering the phase, design, and risk of bias of the contributing trials (e.g., maturity of OS data, interim vs. final analysis).

SECTION 5: DISCUSSION

Item #	PRISMA 2020 Item Description	Location in Manuscript / Response
**23**	**Discussion** - Provide a general interpretation of the results in the context of other evidence, discuss limitations, and implications.	**Section 4 (Discussion):** Includes "4.1 Integration of Major Advances", "4.2 Overarching Themes", "4.3 Clinical Implementation Challenges", "4.4 Limitations of the Evidence Base", and "4.5 Future Research Directions". Covers interpretation, context, limitations, and implications.

SECTION 6: OTHER INFORMATION

Item #	PRISMA 2020 Item Description	Location in Manuscript / Response
**24**	**Registration and protocol** - Provide registration information for the review, including the register name and registration number, or state that the review was not registered.	Section 2.1 and 2.4: The review was conducted according to an a priori protocol. While not registered in PROSPERO due to the 141 rapid evidence synthesis timeline required for contemporary clinical guidance, the review 142 employed rigorous methodological standards throughout the selection, evaluation, and 143 synthesis processes.
**25**	**Support** - Describe sources of financial or non-financial support for the review, and the role of the funders or sponsors in the review.	**Section:** *"Funding"* (in Other Information). States: "This research received no specific grant from any funding agency in the public, commercial, or not-for-profit sectors."

Item #	PRISMA 2020 Item Description	Location in Manuscript / Response
**26**	**Competing interests** - Declare any competing interests of review authors.	**Section:** *"Competing Interests"* (in Other Information). States: "All authors declare that they have no known competing financial interests or personal relationships that could have appeared to influence the work reported in this paper."
**27**	**Data, code and materials availability** - Report which of the following are publicly available and where they can be found: data collection forms; data extracted; analytic code; etc.	**Section:** *"Availability of Data and Materials"* (in Other Information). States that all data are from publicly available sources (conferences, journals) and that the search strategy and data extraction forms are available from the corresponding author upon request.
